# Adaptive lexical processing of semantic competitors extends to alternative names: Evidence from blocked-cyclic picture naming

**DOI:** 10.1177/17470218241245107

**Published:** 2024-04-16

**Authors:** Stefan Wöhner, Andreas Mädebach, Herbert Schriefers, Jörg D. Jescheniak

**Affiliations:** 1 Wilhelm Wundt Institute for Psychology, Leipzig University, Leipzig, Germany; 2 Department of Translation and Language Sciences, Unversitat Pompeu Fabra, Barcelona, Spain; 3 Donders Institute for Brain, Cognition, and Behavior, Radboud University, Nijmegen, The Netherlands

**Keywords:** Word production, blocked-cyclic naming, adaptive processing, taxonomic levels

## Abstract

Naming a picture (e.g., “duck”) in the context of semantically related pictures (e.g., “eagle,” “stork,” “parrot”) takes longer than naming it in the context of unrelated pictures (e.g., “knave,” “toast,” “atlas”). Adaptive models of word production attribute this semantic interference effect in blocked-cyclic naming (BCN) to an adaptive mechanism that makes competitor words, (e.g., the semantically related word “eagle” for the target word “duck”) which are activated but not selected for production, less accessible for future retrieval. Results from a recent picture-word-interference study, however, suggested that alternative names (e.g., “bird” for “duck”) might be exempt from this mechanism, challenging adaptive lexical processing as a general mechanism. We tested whether converging evidence is obtained in BCN. In Experiment 1, we embedded pictures responded to with alternative (category) names (e.g., “bird”) into contexts composed of pictures responded to with specific (exemplar) names (e.g., “duck,” “eagle,” “stork,” and “parrot”). If alternative names are exempt from adaptive lexical processing, interference in the homogeneous context should be found for specific name items but not for alternative name items. In contrast to this prediction, there was similar-sized interference for both types of items. In Experiment 2, we replaced the alternative name items with unrelated items. For these items, interference was largely diminished, ruling out that the effect found in Experiment 1 is a general set effect. Overall, our data suggest that alternative names are not special with respect to adaptive lexical processing.

When a speaker prepares a word for production, a cohort of semantically similar words is activated, resulting from spread of activation in the semantic-lexical network. For example, when the speaker plans to say the word “duck,” also words like “eagle,” “stork,” “bird,” and “mallard” are activated (e.g., Dell, 1986; Howard et al., 2006; Levelt, 1989; Levelt et al., 1991, 1999; Oppenheim et al., 2010; Roelofs, 1992). Consequently, the target word must be selected from this semantic cohort. According to classical word-production models, the activation of non-target competitor words either hinders target word selection (on the lexical selection-by-competition view, e.g., Levelt et al., 1999; Roelofs, 1992, 2018) or has no effect on target word selection (on the non-competitive lexical selection view, e.g., Finkbeiner & Caramazza, 2006). The selection of the target word, however, has no direct effect on the competitor words, neither at the time of selection (except for not being selected) nor at some later point in time. This is different in more recent models of word production (Howard et al., 2006; Oppenheim et al., 2010; see also Damian & Als, 2005). These models incorporate an adaptive mechanism which adjusts the accessibility of both the selected target word and activated but not selected competitor words, after each naming episode.

Adaptive models have been set up to account for the finding of semantic interference in sequential picture naming. There are two task versions, continuous naming (CN) and blocked-cyclic naming (BCN). In standard CN, large sets of pictures are used, and each picture is named only once. In this task version, the naming latency of a picture increases linearly with the number of pictures from this category that have recently been named (e.g., Belke, 2013; Howard et al., 2006). In BCN, small sets of pictures are used, and each picture is named multiple times (one presentation of all pictures of the set is referred to as a cycle). These sets either contain pictures from the same semantic category (homogeneous context) or from different semantic categories (heterogeneous context). In this task version, the naming latency of a picture is longer in the homogeneous context, typically from the second cycle onwards (e.g., Abdel Rahman & Melinger, 2007; Belke, Brysbaert, et al., 2005; Damian & Als, 2005; Damian et al., 2001; Lin et al., 2022; Wöhner et al., 2021).^1^

Adaptive models account for these effects by assuming that after selection of a target word (e.g., “duck”), its accessibility for future retrieval is increased, whereas after non-selection of an activated competitor word (e.g., “stork”), its accessibility for future retrieval is reduced. Oppenheim et al. (2010) related both effects to changes in the connections between semantic features and lexical representations, which are strengthened for the target word (increasing future accessibility) and weakened for competitor words (decreasing future accessibility). Howard et al. (2006) related increased future accessibility of the target word to the strengthening of the connection between a holistic semantic representation and the respective lexical representation and decreased future accessibility of lexical competitors to inhibitory connections between lexical representations.

These mechanisms are assumed to operate uniformly on all words that are activated. That is, if a speaker selects the word “duck” for production, the accessibility of semantically related words from the same taxonomic level, like “eagle” and stork,” but also of words from different taxonomic levels, like “bird,” and “mallard,” should be reduced. Results from a series of picture-word interference (PWI) experiments by Kurtz et al. (2018) seem to question this assumption. Participants named pictures repeatedly (e.g., “duck”). Following the general logic of previous studies (e.g., Jescheniak & Schriefers, 1998; Levelt et al., 1991; Peterson & Savoy, 1998), Kurtz et al. assessed the phonological activation of an alternative name from a different taxonomic level (e.g., “bird” for the target word “duck”) by comparing the effect of a distractor word that is a phonological neighbour of the alternative name (e.g., “birch”) with the effect of an unrelated distractor word (e.g., “mask”). At the beginning of the experiment, there was interference from related distractors (referred to as phonological interference hereafter), indicating that the alternative name was phonologically activated; this replicated previous findings (e.g., Jescheniak et al., 2005, 2017; Jescheniak & Schriefers, 1998; Mädebach et al., 2020, 2022; Peterson & Savoy, 1998). This effect implies that the alternative name was activated at the semantic and abstract-lexical levels as well. Kurtz et al. traced the phonological interference effect across repeated naming. The authors reasoned that if the accessibility of the lexical node of the word “bird” is reduced after non-selection, less activation should be transmitted to its phonological form; hence, phonological interference should be reduced. Contrary to this prediction, phonological interference remained stable over up to about 50 naming episodes in two experiments (see also Wöhner et al., 2023, Experiment 1, for a replication). These results seem to indicate some limitation of the adaptive mechanism, with alternative names being exempt from it. As to the possible functional sense of such a special status, Kurtz et al. referred to the issue of lexical flexibility in spontaneous speech. For example, Almor (1999) pointed out that speakers quite often use different verbal labels as they continue talking about a particular entity. Kurtz et al. reasoned that, under this perspective, effective word selection requires a system that finds a balance between a low probability of mis-selections at a given point in time (and, thus, low accessibility of alternative names) and a high degree of lexical flexibility in the use of different words for the same concept at different points in time (and, thus, high accessibility of alternative names). Low accessibility of alternative names would hinder this. Thus, an adaptive mechanism that does not reduce the accessibility of alternative names is favouring lexical flexibility at increased risk of occasional mis-selection. Levelt et al. (1999) also argued, albeit for a different reason, that appropriate names might be special in terms of lexical processing.

The conclusion drawn by Kurtz et al., however, is complicated by notable methodological differences between their own study and studies that provided data in support of adaptive lexical processing (e.g., Damian & Als, 2005; Howard et al., 2006). Kurtz et al. used the PWI task and focused on the relation between a target word and an alternative name from a different taxonomic level (like “duck” and “bird”). Furthermore, the authors assessed the activation of the alternative name indirectly, by looking at the effect of a distractor word that is a phonological neighbour of the alternative name (like “birch”). In contrast, Damian and Als (2005) and Howard et al. (2006) used either the BCN or the CN task and focused on the relation of a target word and semantic-category coordinates from the same taxonomic level (like “duck” and “stork”). Hence, it is unclear whether the conflicting findings result from the use of different tasks, the testing of different target-competitor relations (see Costa et al., 2003), the indirect versus direct assessment, or any combination of these factors.

This is where the present study ties in. It tested the same target-competitor relation as in PWI experiments of Kurtz et al. (2018) but used the BCN task that has provided evidence for adaptive lexical processing. The critical question was whether—in a homogeneous context made up by pictures of different members of a semantic category which are responded to with their specific names (e.g., “eagle,” “duck,” “stork,” and “parrot”)—the production of the alternative name (viz. category name, “bird”) in response to a generic picture representing that category would be slowed down in the same way as the production of the specific names. If alternative names are exempt from adaptive processing, they should be less affected by the semantic context or be not affected at all. Such an outcome would support the conclusion drawn by Kurtz et al. and be theoretically important because it would provide a challenge to adaptive processing as a general mechanism in word production that operates on all elements of a semantic cohort in the same way. If, by contrast, the production of alternative names is similarly affected by the semantic context, this would call into question the conclusion drawn by Kurtz et al.

There is one previous BCN study by Belke, Meyer, and Damian (2005, Experiment 3) that also asked whether the effect of semantic context established with one set of items extends to novel items. Belke, Meyer, and Damian, however, looked at new items from the same taxonomic level, that is, new semantic-category coordinates, rather than alternative names. In the first four cycles, participants saw a set of items (e.g., four animals [duck, fish, mouse, snake] or four unrelated items [duck, chair, saw, bus]). In the next four cycles, participants either saw a new set of items (e.g., four different animals [ant, frog, owl, pig] or four new unrelated items [ant, shelf, wrench, car]) or the same set of items as before. The authors observed a full generalisation of semantic interference. That is, the interference effect in the homogeneous context in cycles 5 to 8 was of the same size, regardless of whether the items changed or remained the same after cycle 4. Methodologically, this is an elegant procedure for testing the scope of adaptive processing. However, such a procedure depends critically on the possibility to sample a sufficiently large number of new items per semantic category. If one looks for generalisation within a taxonomic level, as Belke, Meyer, and Damian did, this is granted because many categories tend to have numerous exemplars. However, if one looks for generalisation across taxonomic levels, as we do here, the situation is different. In this case, there is only one new item to sample per category, namely the category name. Hence, this situation requires a different procedure than the one used in Belke, Meyer, and Damian et al. (2005). Thus, instead of presenting the two sets of items in separate parts of the experiment, we presented the items interleaved.

## Experiment 1

In Experiment 1, pictures responded to with alternative (category) names (e.g., “bird”) were embedded into contexts composed of pictures responded to with specific (exemplar) names (e.g., “duck,” “eagle,” “stork,” and “parrot”). If alternative names are exempt from adaptive lexical processing, interference in the homogeneous context should be found for specific name items but not for alternative name items.

### Method

#### Participants

We tested 40 participants (34 female, 6 male, *M*_age_ = 24.30 years, *SD*_age_ = 4.57 years, range = 18–34 years), each in three sessions. One participant was replaced because s/he showed up only for the first session. Participants were German native speakers, mostly students from Leipzig University, and had normal or corrected to normal vision. They gave informed written consent before the experiment started and either received course credit or were financially reimbursed (24€). All procedures of our study followed the ethical guidelines of the German Psychological Society (DGPs). According to the regulations of the German Research Council (DFG) that funded our research, the experiment we describe does not require formal approval from a research ethics committee because (a) our method does not involve any risks or emotional or physical stress (participants named pictures of neutral familiar objects), and (b) informed consent was obtained prior to the experiment (see http://www.dfg.de/foerderung/faq/geistes_sozialwissenschaften/index.html).

#### Materials

We selected colour photographs of 60 objects from various sources, four from each of 15 semantic categories. These photographs were used to elicit the production of specific names. We created 15 additional icon-like black line drawings representing these categories; these line drawings were used to elicit the production of alternative (category) names. Pictures were scaled to fill an imaginary square of 250 × 250 pixels (visual angle of about 7.0°× 7.0° at 60-cm distance) and had their background removed. Items were arranged in three 5 × 5 matrices. Each row represented an item subset used for creating a homogeneous block, each column an item subset used for creating a heterogeneous block, see Appendices A1–A3. All subsets derived from one matrix were used in one session. For each subset, a block was created in which pictures were repeated six times, creating six cycles. Blocks involving item subsets in row_*n*_ and column_*n*_ were combined into pairs and presented in direct succession. The sequence of the five pairs was controlled using a sequentially balanced Latin square design. Homogeneous and heterogeneous blocks alternated. Half of the participants started with a homogeneous block and half with a heterogeneous block. The sequence in which the blocks derived from each of the three matrices were presented across sessions was controlled.^2^

The full item set included more specific name items than alternative name items (60 vs. 15); this would lead to more precise point estimates for the former. To avoid this bias, we a priori selected one specific name item per category as a critical item to be included in the data analysis. We based this selection on the results of a pretest that assessed the perceptual similarity of the items. The 15 critical specific name items were selected such that possible differences in perceptual similarity across contexts (homogeneous vs. heterogeneous) between the critical specific name items and the alternative name items were minimised. Twelve participants were tested in the pretest (8 female, 4 male, *M*_age_ = 24.83 years, *SD*_age_ = 5.17 years, range = 19–35 years). On a 5-point Likert-type scale (1 = *visually hardly similar*, 5 = *visually very similar*), they rated all possible combinations of any two items that could be derived from any row or column of the matrices (300 comparisons overall). Because we deliberately tried to keep perceptual similarity among items low during initial item selection, we interspersed 60 filler picture pairs with perceptually highly similar items into the lists to encourage participants to use the full rating scale. In each rating trial, the two items were presented side-by-side on a computer screen until a response was given. For the 30 items to be included in the data analysis (i.e., the 15 critical specific name items and the 15 alternative name items), perceptual similarity ratings were slightly higher in the homogeneous context (*M* = 2.12, *SD* = 0.50, range: 1.21–3.33) than in the heterogeneous context (*M* = 1.49, *SD* = 0.42, range: 1.18–2.63), resembling findings from related studies.^3^ Importantly, this difference was comparable for specific name items (*M*_diff_ = 0.63, *SD*_diff_ = 0.31, range_diff_: −0.03 to 1.25) and alternative name items (*M*_diff_ = 0.63, *SD*_diff_ = 0.32, range_diff_: 0.08–1.08). For more detailed information, see Table S1 in the online Supplemental Material 1 and on the OSF site:https://osf.io/v9yt5/.

#### Apparatus

The experiment was controlled by GNU Octave and the Psychophysics Toolbox (Brainard, 1997; Kleiner et al., 2007; Pelli, 1997), operated under Linux. Pictures were presented on an EIZO S1910 monitor at about 60-cm viewing distance. Responses were registered with a Sennheiser K6/ME64 microphone.

#### Design

Context (homogeneous vs. heterogeneous) and cycle (1–6) were tested within participants and within items. Taxonomic level (specific name vs. alternative name) was tested within participants and between items. There were 900 experimental trials per participant, 360 of which entered the statistical analyses. Forty pseudo-randomised presentation lists per matrix (120 lists overall) were created, each of which was used once. In these lists, direct succession of items with the same phonological onset within a block was excluded.

#### Sample size and power

We used independent data for calculating an estimate of the resulting power of our experiment. For semantic interference in specific name production, the calculation of an estimate of the population effect size was based on ten studies testing young healthy adults with a design comparable to ours (Abdel Rahman & Melinger, 2007, 2011; Belke, 2013; Belke, Brysbaert, et al., 2005; Damian & Als, 2005; Damian et al., 2001; Maess et al., 2002; Navarrete et al., 2012; Wang et al., 2018; Wöhner et al., 2021). The mean standardised effect obtained was *d*_z_ = 1.00, 95% CI [0.76, 1.25] in the participant analysis and *d*_z_ = 0.79, 95% CI [0.64, 0.94] in the item analysis (for details, see https://osf.io/v9yt5/). With 40 participants and 15 items, our experiment had thus a power of >99% in the participant analysis and >80% in the item analysis to observe an effect of this size for specific name production (in a two-sided test with α = .05), when using classical *F*_1_ and *F*_2_ analyses of variance (ANOVAs). For alternative name production, we predicted interference to be absent (in accordance with the results for the phonological interference effect in Kurtz et al.’s PWI experiments), resulting in an interaction of context and taxonomic level. To estimate the power of our design for this interaction, we took the data from Wöhner et al. (2021, Experiment 1, picture naming only, all cycles) for which we could retrieve all parameters needed, including correlations between repeated measures. We estimated power via simulation using the Superpower-package (Lakens & Caldwell, 2022). Cell means entering the simulation were 600 ms (*SD* = 76 ms for participants, *SD* = 20 ms for items) and 581 ms (*SD* = 75 ms for participants, *SD* = 22 ms for items) for specific name production in homogeneous and heterogeneous contexts and 581 ms (*SD* = 75 ms for participants, *SD* = 22 ms for items) for alternative name production in homogeneous and heterogeneous contexts. Correlations between repeated measures were .975 and .663 for participants and items, respectively. The simulation revealed that the design of our experiment (with 40 participants and 15 items) had a power of >99% in the participant analysis and >80% in the item analysis to observe the interaction of context and taxonomic level in an ANOVA (for a two-sided test with α = .05). This analysis shows that the power of our design is sufficiently high, when ANOVAs are used for data analyses as in the previous studies. In the present article, we report results from generalised linear mixed-effects models (GLMMs; for results from corresponding ANOVAs, see https://osf.io/v9yt5/). Assuming that GLMM analyses are at least as sensitive as ANOVAs, the power of our design should be sufficiently high for these analyses as well.

#### Transparency and openness

We report all data exclusions, manipulations, and measures implemented. Our study was not preregistered. We make available all materials, data, and analysis scripts: https://osf.io/v9yt5/. Data analyses were conducted in R (R Core Team, 2023). We used GLMMs as implemented in the lme4-package (Bates et al., 2015, 2023) and the lmerTest-package (Kuznetsova et al., 2017, 2020) with bound optimization by quadratic approximation (“bobyqa”-optimizer) to analyse (nontransformed) naming latencies, assuming a Gamma distribution and an identity link function (Lo & Andrews, 2015). We first conducted analyses with the factors taxonomic level and context, averaged across cycles. To explore whether the development of the context effect across cycles was comparable for specific name responses and alternative name responses, we subsequently conducted corresponding latency analyses for each cycle separately. The variables taxonomic level, context, and their interaction were entered as fixed factors. We used deviation coding (−0.5, 0.5) for specific name responses versus alternative name responses (factor levels of taxonomic level) and heterogeneous context versus homogeneous context (factor levels of context), respectively, to set contrasts for the fixed factors. Participants and items were entered as random factors. Following Barr (2013) and Barr et al. (2013), we started with the maximal random-effects structure given by our design. In each initial model, we included random slopes for all within-unit factors and corresponding interactions. Therefore, we included a by-participant random intercept and random slopes for the two factors context and taxonomic level, and their interaction, and a by-item random intercept and random slope for the factor context. If we encountered a model convergence error, we systematically reduced the complexity of the random-effects structure by stepwise removing random correlations, random slopes for the interaction, and random slopes for main effects until convergence was reached. If there was a singular fit, we excluded random correlations, followed by random slopes that were estimated to be close to zero. However, random slopes for main effects were only excluded after the random slope for the corresponding interaction had been excluded. Once the final random-effects structure was selected, we tested whether correlations between random-effect parameters can be added again without causing a model convergence error or warning message. Model-implied mean naming latencies presented in the tables and figures were computed with the emmeans-package (Lenth, 2023). In addition to these analyses, we performed GLMM analyses on errors, assuming a binomial distribution; linear mixed-effects models (LMMs) on inverse naming latencies (−1000/RT), assuming a normal distribution; and classical *F*_1_ and *F*_2_ ANOVAs on (nontransformed) naming latencies and error proportions using the afex-package (Singmann et al., 2023). These additional analyses lead to the same conclusions. For a full documentation, see https://osf.io//v9yt5/. Our study builds on previous evidence collected in studies with young healthy adults (predominantly university students). We recruited participants from a similar population to ensure comparability with these previous studies.

#### Procedure

Participants were tested individually, each in three sessions. Consecutive sessions were separated by 1–5 days. First, participants received written instructions asking them to respond quickly and accurately. They were then familiarised with the pictures used in the respective session. Each picture was presented on the computer screen for 4 s with its name appearing 500 ms after picture onset below the picture, and participants read the name aloud. In a subsequent practice phase, all pictures were presented once, and participants named them as quickly as possible while avoiding errors. The experimenter corrected the use of non-target names. Finally, the experimental trials were presented with a short break after half of them.

In an experimental trial, the target picture was presented for 1 s in the center of the screen on a light grey background (RGB 220, 220, 220). Naming responses were recorded for 3 s from picture onset and digitally stored on the computer disc. The intertrial interval was 4 s. A session lasted about 45 min.

### Results and discussion

**Figure 1.**
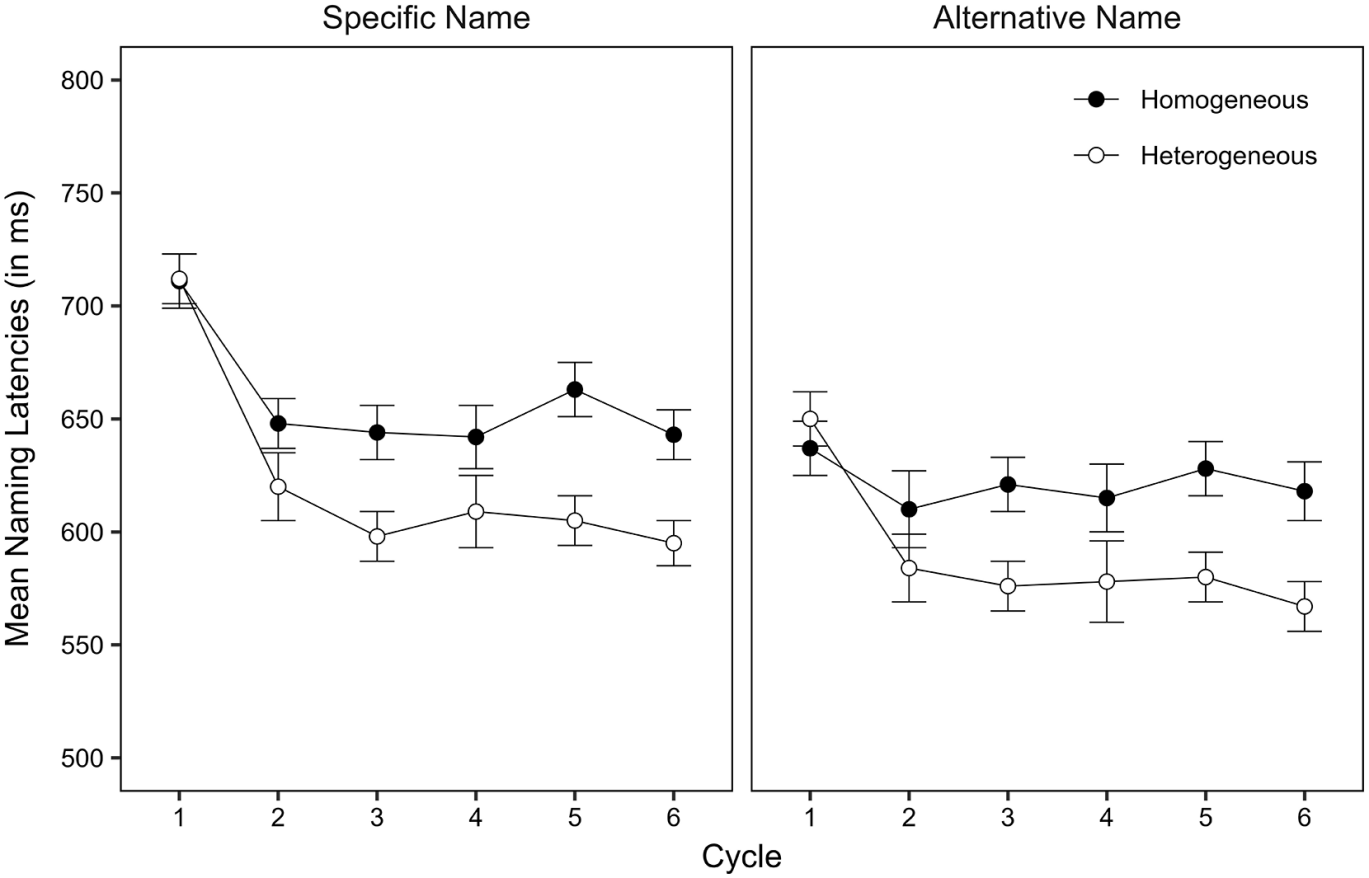
Model implied mean naming latencies for specific name items and alternative name items (in ms) by context and cycle in Experiment 1. Error bars represent the standard error of the mean (SEM).

Naming latencies and naming correctness were determined offline by means of visual and auditory inspection using CheckFiles (Protopapas, 2007). Observations were excluded from the latency analysis and coded as participant errors, if there was a non-expected, missing, or disfluent response (0.66% of all critical observations). Because participant errors were so rare, we do not report their analysis in this article. We should note, though, that there was no indication of a speed-accuracy trade-off. Figure 1 shows model implied mean naming latencies per taxonomic level, context, and cycle. Table 1 shows the fixed-effects estimates. For more detailed information, including statistics on the individual cycles, the error data, inverse naming latencies, and ANOVAs, see https://osf.io/v9yt5/.

**Table 1.** Fixed-effects estimates based on GLMM analyses for naming latencies (in ms) assuming a Gamma distribution and collapsed across cycles from Experiment 1.

Effect	Estimate	*SE*	*z*	*p*
Intercept	622	10	62.26	<.001
Homogeneous—Heterogeneous (C)	33	5	6.99	<.001
Alternative name—Specific name (L)	−36	6	−6.07	<.001
C:L	−3	4	−0.73	.468

*Note*. Formula: RT ~ C + L + C: L + (C + L + C: L || participant) + (C | item). C = semantic context; L = taxonomic level; RT = naming latency.

Naming latencies were slower in the homogeneous context than in the heterogeneous context. They were faster for alternative name responses than for specific name responses, possibly due to the higher lexical frequency of alternative names than of specific names (lemma frequency: 48.22 [*SD* = 50.32] vs. 1.49 [*SD* = 1.62] per million words, type [wordform] frequency: 24.85 [*SD* = 28.68] vs. 0.76 [*SD* = 0.85] per million words; data from dlex, Heister et al., 2011) or due to the easier perceptual analysis and conceptual identification of the line drawings as compared to the photographs. Most importantly, the semantic context effect was similar-sized for specific name responses and alternative name responses. The analysis of the individual cycles revealed that semantic interference was present from cycle 2 onwards and that there was no significant interaction of context with taxonomic level at any of the cycles (see Supplemental Tables S3a–S3g: https://osf.io/v9yt5/).

Experiment 1 showed that specific name responses were slowed down in the homogeneous context from cycle 2 onwards, replicating previous findings. Importantly, such slowing down was found for alternative name items as well. The two interference effects were of comparable size and showed the same development across cycles. This suggests that alternative names were affected by an adaptive mechanism in a similar way as specific names, challenging the conclusion drawn by Kurtz et al. (2018).

However, before accepting this conclusion, an alternative account needs to be considered. One could argue that responses to alternative name items were only slowed down because these items were embedded in the categorically related blocks made up by specific name items. That is, the context effect found for the alternative name items could reflect a general set effect (e.g., modified response criterion or list-composition effect, see Lupker et al., 1997; Meyer et al., 2003; Taylor & Lupker, 2001). Previous studies have indeed shown that when stimuli from various conditions are blocked, speakers may adopt a different criterion for the initiation of a response than when the same stimuli are mixed. That is, a homogeneous context could possibly lead to a general slowdown of alternative name responses, even though their lexical accessibility was not changed by an adaptive mechanism. To the best of our knowledge, in the context of BCN studies, this notion was tested in only one experiment (Damian & Als, 2005, Experiment 2). This experiment found responses for unrelated items to be slower when they were embedded into a homogeneous context than when embedded into a heterogeneous context, but the 9-ms difference was not significant. Notably, the experiment included only eight participants; hence, it likely had insufficient power to allow for any strong conclusion. Therefore, we re-addressed this issue in Experiment 2.

## Experiment 2

In Experiment 2, the alternative (category) name items were replaced with new items that were semantically unrelated in both the homogeneous and the heterogeneous context. Other than that, Experiment 2 was identical to Experiment 1. If semantic interference observed for alternative name items in Experiment 1 reflects a general set effect, it should be replicated in full size for the new unrelated items. In contrast, if it reflects an item-specific adaptive mechanism that operates on co-activated semantic competitors, it should largely disappear.

### Method

#### Participants

Forty participants (32 female, 8 male *M_age_* = 24.18 years, *SD_age_* = 4.15 years, range = 18–39 years) were tested, each in three sessions. One participant was replaced because s/he reported after testing to have been grown up bilingually.

#### Materials

The materials used are same as in Experiment 1, except that the 15 alternative name items were replaced with new items that were unrelated in both the homogeneous and the heterogeneous context; we refer to these items as foils. The foil items were also line drawings and named at the same taxonomic level (see the online Supplemental Material 2: Appendix A1–A3). For the modified item set, we also collected perceptual similarity ratings in a pretest. Based on this pretest (independent sample of 12 participants [10 female, 2 male], *M_age_* = 24.25 years, *SD_age_* = 3.33 years, range = 19–30 years), for each category, we again a priori selected one critical specific name item to be included in the statistical analyses.^4^ For the 30 critical items (15 foil items and 15 selected specific name items), perceptual similarity ratings were higher in the homogeneous context (*M* = 1.98, *SD* = 0.35, range: 1.61–2.77) than in the heterogeneous context (*M* = 1.60, *SD* = 0.34, range: 1.24–2.49), as was the case for the materials used in Experiment 1. Importantly, this difference was comparable for specific name items (*M*_diff_ = 0.42, *SD*_diff_ = 0.46, range_diff_: −0.52 to 1.13) and foil items (*M*_diff_ = 0.34, *SD*_diff_ = 0.14, range_diff_: 0.13–0.60). For more detailed information, see Supplemental Table S7: https://osf.io/v9yt5/.

#### Apparatus and design

Same as in Experiment 1.

#### Power estimate

As in Experiment 1, the critical test was the interaction of level of abstraction and context. The design of Experiment 2 (incl. sample sizes) was identical to the one of Experiment 1. Therefore, the power estimate for the critical interaction was the same as that for Experiment 1 (“Sample size and power” section).

#### Procedure

The procedure followed is same as that in Experiment 1.

### Results and discussion

**Figure 2.**
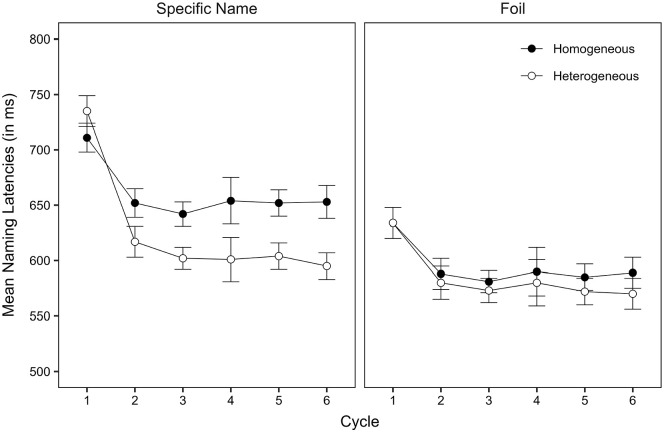
Model implied mean naming latencies for specific name items and foil items (in ms) by context and cycle in Experiment 2. Error bars represent the standard error of the mean (SEM).

The raw data were treated as in Experiment 1. Of all critical observations, 0.88% were coded as participant errors, and 1.85% as technical errors.^5^ Because participant errors were again very rare, we do not report their analysis in this article. As in Experiment 1, there was no indication of a speed-accuracy trade-off. Figure 2 shows model implied mean naming latencies per taxonomic level, context, and cycle. Table 2 shows the fixed-effects estimates. For more detailed information, including statistics on the individual cycles, error data, inverse naming latencies, and ANOVAs, see https://osf.io/v9yt5/.

**Table 2.** Fixed-effects estimates based on GLMM analyses for naming latencies (in ms) assuming a Gamma distribution and collapsed across cycles from Experiment 2.

Effect	Estimate	*SE*	*z*	*p*
Intercept	616	5	135.41	<.001
Homogeneous—Heterogeneous (C)	22	2	9.21	<.001
Foil—Specific name (L)	−54	3	−15.71	<.001
C:L	−28	3	−9.18	<.001

*Note*. Formula: RT ~ C + L + C: L + (C + L + C: L | participant) + (C || item). C = semantic context; L = taxonomic level; RT = naming latency.

Naming latencies were slower in the homogeneous context than in the heterogeneous context. They were faster for foil responses than for specific name responses, again possibly due to the higher lexical frequency of foil names than of specific names (lemma frequency: 16.44 [*SD* = 14.70] vs. 1.97 [*SD* = 1.88] per million words, type [wordform] frequency: 11.54 [*SD* = 10.32] vs. 1.27 [*SD* = 1.44] per million words; data from dlex, Heister et al., 2011) or due to the easier perceptual analysis and conceptual identification of the line drawings than of the photographs. Most importantly, the semantic context effect was substantially smaller for foil responses than for specific name responses. The analysis of the individual cycles revealed that the main effect of context was significant from cycle 2 onwards and that the interaction of context and taxonomic level was significant at each cycle (see Supplemental Tables S9a–S9g: https://osf.io/v9yt5/).

For specific name items, Experiment 2 fully replicated the results from Experiment 1. In fact, the interference effects in the homogeneous context were highly comparable across experiments (36 ms, 95% CI [30, 41] in Experiment 2 vs. 35 ms, 95% CI [26, 43] in Experiment 1). For foil items, there was also an interference effect, but it was substantially smaller than the one for alternative name items in Experiment 1 (8 ms, 95% CI [3, 13] in Experiment 2 vs. 32 ms, 95% CI [20, 43] in Experiment 1). This differential pattern was confirmed in a joint analysis of Experiments 1 and 2, which revealed a significant interaction of context, taxonomic level, and experiment, β = 24, *SE* = 2, *z* = 11.62, *p* < .001 (for more detailed information, see Supplemental Tables S13a–S13c: https://osf.io/v9yt5/). This pattern rules out that responses to alternative name items in Experiment 1 were only slowed down because these items were embedded in the categorically related blocks made up by specific name items. Rather, their slowing down must have a different cause, which is likely reduced lexical accessibility resulting from an adaptive mechanism. The small residual interference effect found with foil items in Experiment 2 possibly reflects a general set effect that also contributes to BCN data patterns. Alternatively, it might result from the slightly higher perceptual similarity of the foil items in homogeneous contexts than in heterogeneous contexts (difference of 0.34 on a 5-point Likert-type scale, see above). However, the development of the effect across cycles does not support this account. If perceptual similarity were the driving force, interference should have been present already in the first cycle (see de Zubicaray et al., 2018). This was not the case, it appeared later.

## General discussion

Using a BCN task, we tested the scope of adaptive processing in word production by looking at semantic interference within a taxonomic level, involving specific name production (of semantic-category coordinates), and between taxonomic levels, involving also alternative name production. Previous BCN studies have consistently shown that picture naming responses are slowed down in a semantically homogeneous context as compared to a semantically heterogeneous context, typically from the second presentation of the pictures onward. The adaptive word production model by Oppenheim et al. (2010; see also Damian & Als, 2005) attributes this interference effect to long-lasting changes in the connection weights between semantic and lexical representations, which occur whenever a word has been selected for production. These changes make activated but non-selected words less accessible for future retrieval.

So far, evidence for such an adaptive mechanism comes from CN and BCN studies looking at the effect, which the recent naming of pictures of semantic-category coordinates exerts on the naming of a target picture (like “eagle” and “stork” for the target “duck”), that is, a situation in which the produced picture names come from the same taxonomic level. The central question of the present study was whether the adaptive mechanism also applies across taxonomic levels, specifically to alternative names of a picture (like “bird” for the target “duck”). We put this question to an experimental test with two BCN experiments. In Experiment 1, generic pictures responded to with alternative (category) names (e.g., “bird”) were interspersed into the context of pictures of category exemplars responded to with specific names (like “eagle” and “stork”) or into the context of pictures of unrelated objects also responded to with specific names (like “toast” and “atlas”). Our central finding is that both specific name production and alternative name production were affected by the semantic context. In fact, both the development and the size of the interference effects were identical. In Experiment 2, the alternative name items were replaced by foil items from the same taxonomic level. While the interference effect for specific name items was of the same size as in Experiment 1, there was only a small residual interference effect for the foil items which likely reflects a general set effect. Its small size suggests that the (full) interference effect for alternative name items in Experiment 1 is not a general set effect but the reflection of a different mechanism, likely an adaptive mechanism reducing the lexical accessibility of these items. Overall, this pattern suggests that the adaptive mechanism applies in a similar way to specific names and to alternative names from a different taxonomic level. Naming the picture of a duck by saying “duck” does not only make words like “eagle” or “stork” less accessible but also an alternative name like “bird.”

However, there are two caveats to this conclusion. The first one relates to a specific property of the BCN task, and the second relates to physical differences between the specific name items and the alternative name items we used. Regarding the first caveat, several authors have suggested that there may be a force at work in BCN that is not present in other production tasks involving picture naming, including PWI and CN. In all these tasks, there are bottom-up effects resulting from the processing of the picture stimulus. As discussed in the introduction part of this article, current production models assume that this leads to an activated cohort of semantically related lexical representations from which the eventual target representation must be selected. In BCN, an additional force may come into play. It has been repeatedly pointed out that BCN involves only a small number of items in each part of the experiment (i.e., in a homogeneous or heterogeneous block, making up a local response set), which are repeatedly presented in discrete cycles before new items are presented (e.g., Belke, 2008, 2013; Belke & Stielow, 2013; Crowther & Martin, 2014). This task feature may be recognised after the first cycle, leading to a task representation that can then become effective. Belke (2008, see also Belke & Stielow, 2013) suggested that this task representation allows participants to effectively top-down bias the items from the local response set for selection (i.e., increase the activation level of their lexical representations). This top-down bias occurs in both the homogeneous context and the heterogeneous context, regardless of whether the lexical items in the respective contexts are connected at the semantic level. However, the top-down bias then interacts with bottom-up effects resulting from the processing of the target picture. According to Belke, such a top-down modulation is efficient in the heterogeneous context because it biases only one lexical representation from each of the semantic categories involved in the local response set. This facilitates the selection of this lexical representation from the cohort of same-category competitors activated bottom-up by the target picture. On the other hand, such a top-down bias is not efficient in the homogeneous context because it biases multiple lexical representations from the same semantic category. This does not facilitate the selection of one lexical representation from the cohort of same-category competitors activated bottom-up by the target picture.

How does this notion of a top-down bias relate to our findings, particularly those from Experiment 1? Experiment 1 showed that specific names and alternative names from a different taxonomic level were inhibited to the same extent by a homogeneous context, suggesting that they were similarly activated by bottom-up processing of the picture input (i.e., a specific name item such as “duck” would activate the lexical representations of specific names such as “duck,” “eagle,” and “stork,” and also, contrary to what Kurtz et al., 2018, have suggested, the lexical representation of an alternative name such as “bird”). The caveat here is perhaps that in our experiment, the alternative name items were part of the local response sets, and thus, their lexical representations were top-down biased in a first step. Given that connections between lexical and semantic representations are thought to be bidirectional (e.g., Dell, 1986; Howard et al., 2006; Levelt et al., 1999; Oppenheim et al., 2010; Roelofs, 1992), this could have led to a complex interaction of top-down and bottom-up effects in the homogeneous context, potentially masking differences in lexical activation that would have resulted from picture-driven bottom-up processing alone. If this is true, the presentation of alternative name items in separate blocks might have resulted in a different pattern.^6^ However, as discussed in the introduction part of this article, this was not possible in the specific case we are dealing with here.

Regarding the second caveat, the specific name items and the alternative name items used in our study differed in terms of stimulus realism (colour photographs vs. black line drawings) and thus physical properties. We chose to do this because it seems impossible to use an identical format for items that elicit naming responses at different taxonomic levels, at least when using a large number of items as we did. On the one hand, there seem to be clear limits to using line drawings to elicit specific naming responses, and in cases where it is possible, the stimuli probably also need to differ in visual properties (more complex for specific name items than for more abstract alternative name items). On the other hand, it seems difficult to use colour photographs of objects to elicit more abstract naming responses, except perhaps when using photographs of rare category exemplars for which participants do not have a specific name. Could this difference in physical properties between item subsets have influenced our pattern of results? As noted earlier, it may have contributed to the observed difference in the overall naming speed for alternative name items and specific name items. However, the critical question addressed in our study is not whether performance on specific name items and alternative name items differs overall—it does, but whether it is differentially affected by context—it is not. Thus, the use of item sets with different physical properties does not undermine the conclusion that the adaptive mechanism applies equally to specific names and to alternative names from a different taxonomic level.

This conclusion, however, conflicts with the one drawn from PWI results by Kurtz et al. (2018). Kurtz et al. observed that specific name production (e.g., “duck”) was slowed down by a distractor word that was a phonological neighbour of the alternative name (e.g., “birch,” related to “bird”) and that this phonological interference effect was stable across many repetitions. Kurtz et al. interpreted the stability of this phonological interference effect as indicating that the accessibility of alternative names is not reduced across repeated naming and, thus, as indicating that alternative names are not subject to an adaptive process.

The contradiction between the conclusions drawn from the present BCN data and the PWI data from Kurtz et al. gets a qualification by recent PWI data and a reanalysis of previous PWI data from Hantsch et al. (2009) by Wöhner et al. (2023). In these experiments, the accessibility of an alternative name was not tested by the phonological interference effect as in Kurtz et al., but rather by a semantic interference effect by using the alternative name itself as distractor (“bird”). In four experiments, semantic interference resulting from these alternative names as distractors was reduced across repeated naming as was semantic interference from category coordinates as distractors (see also Jescheniak et al., 2023, for evidence that the reduction of semantic interference in PWI across repeated naming transfers to new target-distractor combinations). This pattern fully converges with the emergence of interference for alternative name production in BCN, as observed in Experiment 1. Together, these data suggest that, with respect to their accessibility at the abstract-lexical level, alternative names to a target word are subject to an adaptive mechanism in much the same way as are the names of semantic-category coordinates.

This leaves us with the question of how Kurtz et al.’s finding of stable phonological interference in PWI can be reconciled with this conclusion. We can only offer some speculation here. The conclusion by Kurtz et al. critically rests on the assumption that the consequences of the adaptive mechanism on an abstract-lexical level (targeted in BCN and in the use of alternative names as distractors in PWI) map directly and linearly onto the next level of phonological processing, that is, the level at which phonological interference effect of Kurtz et al. is located. However, the validity of this assumption might depend on how activation is transmitted between these processing levels. If the transmission is strongly gated (e.g., Dell & O’Seaghdha, 1991; Rapp & Goldrick, 2000), the consequences of the adaptive mechanism might impact semantic interference—presumably reflecting competition between abstract-lexical representations and phonological interference—presumably reflecting competition between phonological representations, in a different way or to a different degree. While there is some evidence that phonological and orthographic similarity also hampers performance in BCN with pictures (Breining et al., 2016) and CN with words (Mulatti et al., 2012), any comprehensive exploration and evaluation of this and related accounts would require simulations with adaptive computational models that incorporate an abstract-lexical as well as a phonological level of representation and, thus, allow to differentiate between the consequences of adaptive processing at the respective levels. Existing computational adaptive models, however, do not allow to address this question as they are concerned with pre-lexical semantic representations and abstract-lexical representations and changes in connection strength between these levels only (Howard et al., 2006; Oppenheim et al., 2010). Extending these computational models accordingly by including a phonological level seems to be a worthwhile enterprise for future research.

## Conclusion

Using a BCN task, we found comparable semantic interference within taxonomic levels and between taxonomic levels. This pattern suggests that words that are semantically related to a target word, be it a name of a category coordinate from the same taxonomic level or an alternative name from a different taxonomic level, are affected in much the same way by an adaptive mechanism that, across naming episodes, reduces the accessibility of activated but non-selected words. PWI data by Kurtz et al. (2018) had suggested that alternative names to a target word are exempt from such changes, indicating a limitation of the adaptive mechanism. The present BCN data, however, along with recent independent PWI data by Wöhner et al. (2023), do not support this claim. Rather, they suggest that adaptive processing applies broadly to all semantically related and thus co-activated words.

## Supplemental Material

sj-docx-1-qjp-10.1177_17470218241245107 – Supplemental material for Adaptive lexical processing of semantic competitors extends to alternative names: Evidence from blocked-cyclic picture naming Supplemental material, sj-docx-1-qjp-10.1177_17470218241245107 for Adaptive lexical processing of semantic competitors extends to alternative names: Evidence from blocked-cyclic picture naming by Stefan Wöhner, Andreas Mädebach, Herbert Schriefers and Jörg D. Jescheniak in Quarterly Journal of Experimental Psychology

## Notes

1. There is some discussion of why the interference effect in BCN, other than the interference effect in CN, is not cumulative, that is, does not increase after the second cycle (e.g., Belke & Stielow, 2013). For the present study, this is not a critical issue as long as one assumes that the rise of interference from the first to the second cycle in BCN reflects some kind of adaptive process (as is proposed, e.g., by Belke, 2013; Damian & Als, 2005; Oppenheim et al., 2010; Roelofs, 2018). What is critical for the present study is whether specific name production and alternative name production yield comparable or different patterns of interference.

2. Full sequential counterbalancing of all factors, including the assignment of material subsets (i.e., items from the different matrices) to sessions, would have required 60 participants (or a multiple thereof), which must be considered a needlessly large participant sample for a BCN experiment (see power estimates). As we did not expect systematic effects of item subsets or their sequence of presentation, we only took care that each subset of items was tested about equally often during each of the three sessions and that the sequence in which the material subsets were tested was sequentially controlled as well as possible with our sample.

3. For example, Wöhner et al. (2021) reported a difference of 0.50, and Damian et al. (2001), a difference of 0.48 (same scale as used here).

4. The subsets of critical specific name items differed in Experiments 1 and 2 because they were selected against the standard of different item sets (alternative name or foil).

5. Due to apparatus failure, the audio recordings from one session were lost for two participants, so that no naming latencies could be determined offline for the respective trials.

6. We thank one of the reviewers for bringing this to our attention.
