# Supplementary material for: Adaptive lexical processing of semantic competitors extends to alternative names: Evidence from blocked-cyclic picture naming
Source: Q J Exp Psychol (Hove). 2024 Apr 16;78(4):672–84. doi: 10.1177/17470218241245107 (PMC11905327; doi:10.1177/17470218241245107)
Supplement: sj-docx-1-qjp-10.1177_17470218241245107 – Supplemental material for Adaptive lexical processing of semantic competitors extends to alternative names: Evidence from blocked-cyclic picture naming [file sj-docx-1-qjp-10.1177_17470218241245107.docx]

Supplementary Material for:

Adaptive Lexical Processing of Semantic Competitors Extends
to Alternative Names: Evidence from Blocked-Cyclic Picture Naming

Stefan Wöhner^1^, Andreas Mädebach^2,1^, Herbert Schriefers^3^, & Jörg D. Jescheniak^1^

^1^ Leipzig University, ^2^ Universitat Pompeu Fabra, ^3^ Radboud University

**Data Availability Statement**

All materials, data, and analysis scripts are stored in an open science framework (OSF) archive and are freely available at <https://osf.io/v9yt5/>.

**SUPPLEMENTARY MATERIAL 1**

**EXPERIMENT 1**

**RESULTS FROM PERCEPTUAL SIMILARITY RATINGS**

Table S1

*Mean Rating Scores by Taxonomic Level and Context from Experiment 1.*

| Context | *M* | *SD* | *SE* | Minimum | Maximum |
| --- | --- | --- | --- | --- | --- |
|  | Alternative Name Items (*N* = 15) | | | | |
| Homogeneous | 2.03 | 0.52 | 0.15 | 1.28 | 3.33 |
| Heterogeneous | 1.40 | 0.37 | 0.11 | 1.13 | 2.50 |
| Difference | 0.63 | 0.32 | 0.09 | 0.08 | 1.08 |
|  | Critical Specific Name Items (*N* = 15) | | | | |
| Homogeneous | 2.22 | 0.55 | 0.16 | 1.13 | 3.32 |
| Heterogeneous | 1.58 | 0.50 | 0.14 | 1.17 | 2.75 |
| Difference | 0.63 | 0.31 | 0.09 | -0.03 | 1.25 |
|  | Noncritical Specific Name Items (*N* = 45) | | | | |
| Homogeneous | 2.26 | 0.53 | 0.15 | 1.27 | 3.36 |
| Heterogeneous | 1.61 | 0.47 | 0.13 | 1.25 | 2.71 |
| Difference | 0.66 | 0.30 | 0.09 | -0.02 | 1.28 |
|  | Alternative Name (*N* = 15) and Critical Specific Name Items (*N* = 15) | | | | |
| Homogeneous | 2.12 | 0.50 | 0.14 | 1.21 | 3.33 |
| Heterogeneous | 1.49 | 0.42 | 0.12 | 1.18 | 2.63 |
| Difference | 0.63 | 0.29 | 0.08 | 0.03 | 1.15 |

*Note.* Values on 5-point Likert scale. 1 = visually hardly similar; 5 = visually very similar.

**RESULTS FROM GLMM ANALYSES ON NONTRANSFORMED NAMING LATENCIES AND ERRORS (COMPLEMENTING THE ANALYSES REPORTED IN THE ARTICLE)**

Table S2a

*Model Implied Mean Naming Latencies (in ms) by Taxonomic Level, Context, and Cycle from Experiment 1. The Standard Error of the Mean is Given in Parentheses.*

| Context | Cycle | | | | | |  | Overall |
| --- | --- | --- | --- | --- | --- | --- | --- | --- |
|  | C_1_ | C_2_ | C_3_ | C_4_ | C_5_ | C_6_ |  | C_1_ – C_6_ |
| Specific Name | | | | | | | | |
| Homogeneous | 711 (12) | 648 (11) | 644 (12) | 642 (14) | 663 (12) | 643 (11) |  | 657 (13) |
| Heterogeneous | 712 (11) | 620 (15) | 598 (11) | 609 (16) | 605 (11) | 595 (10) |  | 622 (12) |
| Difference | -1 (10) | 28 (14) | 46 (8) | 33 (9) | 58 (9) | 48 (8) |  | 35 (4) |
| 95% CI | [-20; 17] | [0; 56] | [31; 61] | [14; 51] | [40; 75] | [32; 63] |  | [26; 43] |
| Alternative Name | | | | | | | | |
| Homogeneous | 637 (12) | 610 (17) | 621 (12) | 615 (15) | 628 (12) | 618 (13) |  | 620 (9) |
| Heterogeneous | 650 (12) | 584 (15) | 576 (11) | 578 (18) | 580 (11) | 567 (11) |  | 588 (7) |
| Difference | -13 (9) | 26 (14) | 46 (8) | 37 (9) | 47 (9) | 52 (9) |  | 32 (6) |
| 95% CI | [-31; 6] | [-3; 54] | [30; 61] | [19; 56] | [31; 64] | [35; 69] |  | [20; 43] |

*Note.* CI = confidence interval.

Table S2b

*Model Implied Errors (in Log Odds) by Taxonomic Level, Context, and Cycle from Experiment 1. The Standard Error of the Mean is Given in Parentheses.*

| Context | Cycle | | | | | |  | Overall |
| --- | --- | --- | --- | --- | --- | --- | --- | --- |
|  | C_1_ | C_2_ | C_3_ | C_4_ | C_5_ | C_6_ |  | C_1_ – C_6_ |
| Specific Name | | | | | | | | |
| Homogeneous | -5.97 (1.00) | -9.29 (1.68) | -6.25 (0.92) | -4.20 (0.56) | -8.00 (3.39) | -6.99 (1.16) |  | -5.05 (0.31) |
| Heterogeneous | -4.42 (0.55) | -15.61 (3.76) | -6.48 (0.96) | -6.62 (1.11) | -10.01 (3.85) | -7.69 (1.36) |  | -6.03 (0.49) |
| Difference | -1.55 (1.11) | 6.33 (3.14) | 0.24 (0.99) | 2.42 (1.05) | 2.01 (1.53) | 0.70 (1.23) |  | 0.98 (0.55) |
| 95% CI | [-3.73; 0.62] | [0.18; 12.47] | [-1.71; 2.18] | [0.37; 4.47] | [-0.98; 5.00] | [-1.71; 3.12] |  | [-0.11; 2.06] |
| Alternative Name | | | | | | | | |
| Homogeneous | -6.40 (1.05) | -10.75 (1.90) | -6.06 (0.91) | -5.00 (0.64) | -8.47 (3.62) | -5.87 (0.99) |  | -5.35 (0.33) |
| Heterogeneous | -5.45 (0.69) | -16.34 (4.03) | -7.22 (1.09) | -6.62 (1.10) | -28.96 (148.00) | -24.34 (63.58) |  | -6.99 (0.59) |
| Difference | -0.95 (1.22) | 5.60 (3.55) | 1.15 (1.13) | 1.62 (1.10) | 20.49 (148.07) | 18.46 (63.58) |  | 1.65 (0.66) |
| 95% CI | [-3.34; 1.45] | [-1.37; 12.56] | [-1.06; 3.36] | [-0.53; 3.77] | [-269.73; 310.71] | [-106.15; 143.08] |  | [0.36; 2.94] |

*Note.* CI = confidence interval.

Table S3a

*Fixed Effects Estimates Based on GLMM Analyses for Naming Latencies (Nontransformed, Assuming a Gamma Distribution) and for Errors (Assuming a Binomial Distribution) across all Cycles from Experiment 1.*

|  | Naming Latencies | | | | | Errors | | | |
| --- | --- | --- | --- | --- | --- | --- | --- | --- | --- |
| Effect | Estimate | *SE* | *z* | *p* | Estimate | | *SE* | *z* | *p* |
| Intercept | 622 | 10 | 62.26 | < .001 | -5.85 | | 0.28 | -20.61 | < .001 |
| Homogeneous – Heterogeneous (C) | 33 | 5 | 6.99 | < .001 | 1.31 | | 0.53 | 2.45 | .014 |
| Alternative Name – Specific Name  (L) | -36 | 6 | -6.07 | < .001 | -0.63 | | 0.33 | -1.92 | .055 |
| C:L | -3 | 4 | -0.73 | .468 | 0.67 | | 0.58 | 1.15 | .249 |

*Note*. Formula for the analysis of naming latencies: RT ~ C + L + C:L + (C + L + C:L || participant) + (C | item). Formula for the analysis of errors: ER ~ C + L + C:L + (C | participant) + (C | item). C = semantic context; L = taxonomic level; RT = naming latency; ER = error.

Table S3b

*Fixed Effects Estimates Based on GLMM Analyses for Naming Latencies (Nontransformed, Assuming a Gamma Distribution) and for Errors (Assuming a Binomial Distribution) for Cycle 1 from Experiment 1.*

|  | Naming Latencies | | | | | Errors | | | |
| --- | --- | --- | --- | --- | --- | --- | --- | --- | --- |
| Effect | Estimate | *SE* | *z* | *p* | Estimate | | *SE* | *z* | *p* |
| Intercept | 678 | 9 | 75.92 | < .001 | -5.56 | | 0.57 | -9.77 | < .001 |
| Homogeneous – Heterogeneous (C) | -7 | 8 | -0.87 | .386 | -1.25 | | 1.05 | -1.19 | .235 |
| Alternative Name – Specific Name  (L) | -68 | 12 | -5.88 | < .001 | -0.73 | | 0.45 | -1.60 | .109 |
| C:L | -11 | 9 | -1.25 | .210 | 0.60 | | 1.01 | 0.60 | .549 |

*Note*. Formula for the analysis of naming latencies: RT ~ C + L + C:L + (C + L + C:L | participant) + (C || item). Formula for the analysis of errors: ER ~ C + L + C:L + (C | participant) + (C || item). C = semantic context; L = taxonomic level; RT = naming latency; ER = error.

Table S3c

*Fixed Effects Estimates Based on GLMM Analyses for Naming Latencies (Nontransformed, Assuming a Gamma Distribution) and for Errors (Assuming a Binomial Distribution) for Cycle 2 from Experiment 1.*

|  | Naming Latencies | | | | | Errors | | | |
| --- | --- | --- | --- | --- | --- | --- | --- | --- | --- |
| Effect | Estimate | *SE* | *z* | *p* | Estimate | | *SE* | *z* | *p* |
| Intercept | 615 | 12 | 53.28 | < .001 | -13.00 | | 2.40 | -5.41 | < .001 |
| Homogeneous – Heterogeneous (C) | 27 | 11 | 2.45 | .014 | 5.96 | | 2.95 | 2.02 | .043 |
| Alternative Name – Specific Name  (L) | -37 | 11 | -3.45 | .001 | -1.10 | | 1.58 | -0.70 | .487 |
| C:L | -2 | 18 | -0.13 | .894 | -0.73 | | 3.18 | -0.23 | .819 |

*Note*. Formula for the analysis of naming latencies: RT ~ C + L + C:L + (C + L + C:L | participant) + (C || item). Formula for the analysis of errors: ER ~ C + L + C:L + (C || participant) + (C | item). C = semantic context; L = taxonomic level; RT = naming latency; ER = error.

Table S3d

*Fixed Effects Estimates Based on GLMM Analyses for Naming Latencies (Nontransformed, Assuming a Gamma Distribution) and for Errors (Assuming a Binomial Distribution) for Cycle 3 from Experiment 1.*

|  | Naming Latencies | | | | | Errors | | | |
| --- | --- | --- | --- | --- | --- | --- | --- | --- | --- |
| Effect | Estimate | *SE* | *z* | *p* | Estimate | | *SE* | *z* | *p* |
| Intercept | 610 | 10 | 61.52 | < .001 | -6.50 | | 0.75 | -8.69 | < .001 |
| Homogeneous – Heterogeneous (C) | 46 | 6 | 7.24 | < .001 | 0.69 | | 0.81 | 0.85 | .393 |
| Alternative Name – Specific Name  (L) | -23 | 8 | -2.92 | .004 | -0.27 | | 0.64 | -0.43 | .668 |
| C:L | -1 | 8 | -0.08 | .934 | 0.91 | | 1.36 | 0.67 | .503 |

*Note*. Formula for the analysis of naming latencies: RT ~ C + L + C:L + (C + L + C:L || participant) + (C | item). Formula for the analysis of errors: ER ~ C + L + C:L + (C + L || participant) + (C || item). C = semantic context; L = taxonomic level; RT = naming latency; ER = error.

Table S3e

*Fixed Effects Estimates Based on GLMM Analyses for Naming Latencies (Nontransformed, Assuming a Gamma Distribution) and for Errors (Assuming a Binomial Distribution) for Cycle 4 from Experiment 1.*

|  | Naming Latencies | | | | | Errors | | | |
| --- | --- | --- | --- | --- | --- | --- | --- | --- | --- |
| Effect | Estimate | *SE* | *z* | *p* | Estimate | | *SE* | *z* | *p* |
| Intercept | 611 | 14 | 42.46 | < .001 | -5.61 | | 0.60 | -9.38 | < .001 |
| Homogeneous – Heterogeneous (C) | 35 | 9 | 4.11 | < .001 | 2.02 | | 0.76 | 2.66 | .008 |
| Alternative Name – Specific Name  (L) | -29 | 9 | -3.33 | .001 | -0.40 | | 0.78 | -0.51 | .611 |
| C:L | 4 | 8 | 0.59 | .555 | -0.80 | | 1.52 | -0.53 | .597 |

*Note*. Formula for the analysis of naming latencies: RT ~ C + L + C:L + (C + L + C:L | participant) + (C | item). Formula for the analysis of errors: ER ~ C + L + C:L + (1 | participant) + (1 | item). C = semantic context; L = taxonomic level; RT = naming latency; ER = error.

Table S3f

*Fixed Effects Estimates Based on GLMM Analyses for Naming Latencies (Nontransformed, Assuming a Gamma Distribution) and for Errors (Assuming a Binomial Distribution) for Cycle 5 from Experiment 1.*

|  | Naming Latencies | | | | | Errors | | | |
| --- | --- | --- | --- | --- | --- | --- | --- | --- | --- |
| Effect | Estimate | *SE* | *z* | *p* | Estimate | | *SE* | *z* | *p* |
| Intercept | 619 | 10 | 64.28 | < .001 | -13.86 | | 37.10 | -0.37 | .709 |
| Homogeneous – Heterogeneous (C) | 53 | 8 | 6.66 | < .001 | 11.25 | | 74.05 | 0.15 | .879 |
| Alternative Name – Specific Name  (L) | -30 | 8 | -3.69 | < .001 | -9.71 | | 73.99 | -0.13 | .896 |
| C:L | -10 | 8 | -1.30 | .195 | 18.48 | | 148.07 | 0.12 | .901 |

*Note*. Formula for the analysis of naming latencies: RT ~ C + L + C:L + (C + L + C:L | participant) + (C | item). Formula for the analysis of errors: ER ~ C + L + C:L + (L | participant) + (C || item). C = semantic context; L = taxonomic level; RT = naming latency; ER = error.

Table S3g

*Fixed Effects Estimates Based on GLMM Analyses for Naming Latencies (Nontransformed, Assuming a Gamma Distribution) and for Errors (Assuming a Binomial Distribution) for Cycle 6 from Experiment 1.*

|  | Naming Latencies | | | | | Errors | | | |
| --- | --- | --- | --- | --- | --- | --- | --- | --- | --- |
| Effect | Estimate | *SE* | *z* | *p* | Estimate | | *SE* | *z* | *p* |
| Intercept | 606 | 10 | 61.97 | < .001 | -11.22 | | 15.92 | -0.70 | .481 |
| Homogeneous – Heterogeneous (C) | 50 | 7 | 7.36 | < .001 | 9.58 | | 31.80 | 0.30 | .763 |
| Alternative Name – Specific Name  (L) | -27 | 9 | -3.10 | .002 | -7.77 | | 31.80 | -0.24 | .807 |
| C:L | 4 | 10 | 0.41 | .681 | 17.76 | | 63.58 | 0.28 | .780 |

*Note*. Formula for the analysis of naming latencies: RT ~ C + L + C:L + (C + L + C:L | participant) + (C | item). Formula for the analysis of errors: ER ~ C + L + C:L + (1 | participant) + (1 | item). C = semantic context; L = taxonomic level; RT = naming latency; ER = error.

**RESULTS FROM LMM ANALYSES ON INVERSE NAMING LATENCIES (ADDITIONAL ANALYSES)**

Table S4a

*Fixed Effects Estimates Based on LMM Analyses for Inverse Naming Latencies (- 1000/RT, Assuming a Normal Distribution) across all Cycles from Experiment 1.*

| Effect | Estimate | *SE* | *df* | *t* | *p* |
| --- | --- | --- | --- | --- | --- |
| Intercept | -1.71 | 0.03 | 49.91 | -59.62 | < .001 |
| Homogeneous – Heterogeneous (C) | 0.08 | 0.01 | 41.19 | 8.11 | < .001 |
| Alternative Name – Specific Name  (L) | -0.10 | 0.02 | 31.51 | -4.93 | < .001 |
| C:L | < 0.01 | 0.02 | 31.31 | -0.18 | .858 |

*Note*. Formula: RT ~ C + L + C:L + (C + L + C:L | participant) + (C | item). C = semantic context; L = taxonomic level; RT = inverse naming latency.

Table S4b

*Fixed Effects Estimates Based on LMM Analyses for Inverse Naming Latencies (- 1000/RT, Assuming a Normal Distribution) for Cycle 1 from Experiment 1.*

| Effect | Estimate | *SE* | *df* | *t* | *p* |
| --- | --- | --- | --- | --- | --- |
| Intercept | -1.58 | 0.03 | 54.96 | -61.10 | < .001 |
| Homogeneous – Heterogeneous (C) | -0.05 | 0.02 | 38.51 | -2.53 | .016 |
| Alternative Name – Specific Name  (L) | -0.17 | 0.03 | 33.91 | -6.28 | < .001 |
| C:L | -0.02 | 0.04 | 41.33 | -0.48 | .637 |

*Note*. Formula: RT ~ C + L + C:L + (C + L + C:L || participant) + (C | item). C = semantic context; L = taxonomic level; RT = inverse naming latency.

Table S4c

*Fixed Effects Estimates Based on LMM Analyses for Inverse Naming Latencies (- 1000/RT, Assuming a Normal Distribution) for Cycle 2 from Experiment 1.*

| Effect | Estimate | *SE* | *df* | *t* | *p* |
| --- | --- | --- | --- | --- | --- |
| Intercept | -1.73 | 0.03 | 46.97 | -57.39 | < .001 |
| Homogeneous – Heterogeneous (C) | 0.07 | 0.02 | 30.20 | 3.98 | < .001 |
| Alternative Name – Specific Name  (L) | -0.10 | 0.02 | 29.26 | -4.93 | < .001 |
| C:L | < 0.01 | 0.03 | 29.91 | -0.13 | .898 |

*Note*. Formula: RT ~ C + L + C:L + (C | participant) + (C | item). C = semantic context; L = taxonomic level; RT = inverse naming latency.

Table S4d

*Fixed Effects Estimates Based on LMM Analyses for Inverse Naming Latencies (- 1000/RT, Assuming a Normal Distribution) for Cycle 3 from Experiment 1.*

| Effect | Estimate | *SE* | *df* | *t* | *p* |
| --- | --- | --- | --- | --- | --- |
| Intercept | -1.74 | 0.03 | 49.42 | -58.75 | < .001 |
| Homogeneous – Heterogeneous (C) | 0.12 | 0.01 | 24.95 | 8.96 | < .001 |
| Alternative Name – Specific Name  (L) | -0.07 | 0.02 | 29.27 | -3.14 | .004 |
| C:L | < 0.01 | 0.02 | 29.85 | -0.04 | .969 |

*Note*. Formula for the analysis of inverse naming latencies: RT ~ C + L + C:L + (C | participant) + (C | item). C = semantic context; L = taxonomic level; RT = inverse naming latency.

Table S4e

*Fixed Effects Estimates Based on LMM Analyses for Inverse Naming Latencies (- 1000/RT, Assuming a Normal Distribution) for Cycle 4 from Experiment 1.*

| Effect | Estimate | *SE* | *df* | *t* | *p* |
| --- | --- | --- | --- | --- | --- |
| Intercept | -1.74 | 0.03 | 47.58 | -56.06 | < .001 |
| Homogeneous – Heterogeneous (C) | 0.09 | 0.01 | 27.10 | 6.19 | < .001 |
| Alternative Name – Specific Name  (L) | -0.09 | 0.02 | 29.25 | -4.02 | < .001 |
| C:L | 0.02 | 0.03 | 30.04 | 0.76 | .455 |

*Note*. Formula: RT ~ C + L + C:L + (C | participant) + (C | item). C = semantic context; L = taxonomic level; RT = inverse naming latency.

Table S4f

*Fixed Effects Estimates Based on LMM Analyses for Inverse Naming Latencies (- 1000/RT, Assuming a Normal Distribution) for Cycle 5 from Experiment 1.*

| Effect | Estimate | *SE* | *df* | *t* | *p* |
| --- | --- | --- | --- | --- | --- |
| Intercept | -1.73 | 0.03 | 49.19 | -56.97 | < .001 |
| Homogeneous – Heterogeneous (C) | 0.13 | 0.02 | 33.81 | 7.57 | < .001 |
| Alternative Name – Specific Name  (L) | -0.10 | 0.02 | 30.05 | -4.16 | < .001 |
| C:L | -0.02 | 0.03 | 29.99 | -0.78 | .444 |

*Note*. Formula: RT ~ C + L + C:L + (C + L || participant) + (C | item). C = semantic context; L = taxonomic level; RT = inverse naming latency.

Table S4g

*Fixed Effects Estimates Based on LMM Analyses for Inverse Naming Latencies (- 1000/RT, Assuming a Normal Distribution) for Cycle 6 from Experiment 1.*

| Effect | Estimate | *SE* | *df* | *t* | *p* |
| --- | --- | --- | --- | --- | --- |
| Intercept | -1.75 | 0.03 | 49.43 | -58.78 | < .001 |
| Homogeneous – Heterogeneous (C) | 0.13 | 0.01 | 29.90 | 9.48 | < .001 |
| Alternative Name – Specific Name  (L) | -0.08 | 0.02 | 29.34 | -3.37 | .002 |
| C:L | 0.01 | 0.03 | 29.90 | 0.44 | .661 |

*Note*. Formula: RT ~ C + L + C:L + (1 | participant) + (C | item). C = semantic context; L = taxonomic level; RT = inverse naming latency.

**RESULTS FROM ANOVAS ON NAMING LATENCIES AND ERROR PROPORTIONS (ADDITIONAL ANALYSES)**

Table S5a

*Mean Naming Latencies (in ms) by Taxonomic Level, Context, and Cycle from Experiment 1. The Standard Error of the Mean is Given in Parentheses.*

| Context | Cycle | | | | | |  | Overall |
| --- | --- | --- | --- | --- | --- | --- | --- | --- |
|  | C_1_ | C_2_ | C_3_ | C_4_ | C_5_ | C_6_ |  | C_1_ – C_6_ |
| Specific Name | | | | | | | | |
| Homogeneous | 693  (14) | 635  (12) | 632  (12) | 628  (12) | 648  (12) | 631  (12) |  | 644  (12) |
| Heterogeneous | 698  (12) | 606  (13) | 587  (12) | 595  (13) | 593  (12) | 585  (11) |  | 611  (11) |
| Difference | -5  (8) | 29  (7) | 45  (6) | 33  (6) | 55  (11) | 46  (6) |  | 34  (4) |
| 95% CI | [-20; 10] | [16; 43] | [34; 57] | [21; 44] | [33; 77] | [33; 58] |  | [27; 41] |
| Alternative Name | | | | | | | | |
| Homogeneous | 619  (13) | 597  (11) | 608  (12) | 601  (12) | 611  (14) | 606  (11) |  | 607  (11) |
| Heterogeneous | 635  (12) | 573  (10) | 563  (10) | 564  (13) | 566  (12) | 557  (10) |  | 576  (10) |
| Difference | -15  (16) | 25  (6) | 45  (7) | 37  (6) | 44  (6) | 49  (6) |  | 31  (4) |
| 95% CI | [-47; 16] | [12; 37] | [30; 60] | [25; 50] | [32; 57] | [36; 61] |  | [23; 39] |

*Note.* CI = confidence interval. Naming latencies are based on aggregated participant data.

Table S5b

*Mean Error proportions (in %) by Taxonomic Level, Context, and Cycle from Experiment 1. The Standard Error of the Mean is Given in Parentheses.*

| Context | Cycle | | | | | |  | Overall |
| --- | --- | --- | --- | --- | --- | --- | --- | --- |
|  | C_1_ | C_2_ | C_3_ | C_4_ | C_5_ | C_6_ |  | C_1_ – C_6_ |
| Specific Name | | | | | | | | |
| Homogeneous | 1.00  (0.45) | 1.17  (0.53) | 0.83  (0.35) | 1.83  (0.53) | 1.17  (0.47) | 0.33  (0.23) |  | 1.06  (0.22) |
| Heterogeneous | 1.83  (0.53) | 0.33  (0.23) | 0.67  (0.40) | 0.17  (0.17) | 0.17  (0.17) | 0.17  (0.17) |  | 0.56  (0.14) |
| Difference | -0.83  (0.64) | 0.83  (0.59) | 0.17  (0.56) | 1.67  (0.57) | 1.00  (0.45) | 0.17  (0.29) |  | 0.50  (0.25) |
| 95% CI | [-2.13; 0.46] | [-0.37; 2.03] | [-0.96; 1.30] | [0.51; 2.82] | [0.09; 1.91] | [-0.42; 0.76] |  | [0.00; 1.00] |
| Alternative Name | | | | | | | | |
| Homogeneous | 0.67  (0.32) | 0.33  (0.23) | 1.00  (0.38) | 0.83  (0.35) | 1.00  (0.45) | 1.00  (0.56) |  | 0.81  (0.18) |
| Heterogeneous | 0.67  (0.32) | 0.17  (0.17) | 0.33  (0.23) | 0.17  (0.17) | 0.00  (0.00) | 0.00  (0.00) |  | 0.22  (0.10) |
| Difference | 0.00  (0.48) | 0.17  (0.29) | 0.67  (0.47) | 0.67  (0.40) | 1.00  (0.45) | 1.00  (0.56) |  | 0.58  (0.21) |
| 95% CI | [-0.97; 0.97] | [-0.42; 0.76] | [-0.27; 1.61] | [-0.14; 1.47] | [0.09; 1.91] | [-0.14; 2.14] |  | [0.16; 1.01] |

*Note.* CI = confidence interval. Error proportions are based on aggregated participant data.

Table S6

*Analyses of Variance for Naming Latencies and Error Proportions including factors Context, Cycle, and Taxonomic Level from Experiment 1.*

| Participant Analysis | | | | | Item Analysis | | | | |
| --- | --- | --- | --- | --- | --- | --- | --- | --- | --- |
| Effect | df | *F* | *p* | η_G_^2^ | Effect | df | *F* | *p* | η_G_^2^ |
| Naming Latencies | | | | | | | | | |
| C | 1, 39 | 116.98 | < .001 | .045 | C | 1, 28 | 84.26 | < .001 | .233 |
| Cy | 3.56, 138.86 | 83.64 | < .001 | .088 | Cy | 2.50, 70.01 | 70.72 | < .001 | .388 |
| C:Cy | 3.33, 130.01 | 17.05 | < .001 | .019 | C:Cy | 5, 140 | 19.14 | < .001 | .114 |
| L | 1, 39 | 186.88 | < .001 | .054 | L | 1, 28 | 23.07 | < .001 | .275 |
| C:L | 1, 39 | 0.45 | .507 | < .001 | C:L | 1, 28 | 0.21 | .651 | .001 |
| Cy:L | 3.56, 138.86 | 11.94 | < .001 | .010 | Cy:L | 2.50, 70.01 | 7.32 | .001 | .062 |
| C:Cy:L | 2.84, 110.65 | 0.35 | .780 | < .001 | C:Cy:L | 5, 140 | 0.38 | .864 | .003 |
| Error Proportions | | | | | | | | | |
| C | 1, 39 | 8.67 | .005 | .015 | C | 1, 28 | 10.92 | .003 | .038 |
| Cy | 3.93, 153.17 | 1.91 | .112 | .009 | Cy | 5, 140 | 2.13 | .065 | .024 |
| C:Cy | 5, 195 | 2.68 | .023 | .013 | C:Cy | 3.39, 94.83 | 2.25 | .080 | .033 |
| L | 1, 39 | 5.00 | .031 | .004 | L | 1, 28 | 2.13 | .156 | .011 |
| C:L | 1, 39 | 0.09 | .760 | < .001 | C:L | 1, 28 | 0.06 | .801 | < .001 |
| Cy:L | 5, 195 | 1.22 | .299 | .006 | Cy:L | 5, 140 | 1.29 | .274 | .014 |
| C:Cy:L | 5, 195 | 1.36 | .243 | .007 | C:Cy:L | 3.39, 94.83 | 1.11 | .351 | .017 |

*Note.* Corrected degrees of freedom (df) and *p*-values are reported (based on Greenhouse-Geisser correction), if the sphericity assumption is violated (Mauchly's test: *p* < .05).C = semantic context; Cy = cycle; L = taxonomic level.

**EXPERIMENT 2**

**RESULTS FROM PERCEPTUAL SIMILARITY RATINGS**

Table S7

*Mean Rating Scores by Taxonomic Level and Context from Experiment 2.*

| Context | *M* | *SD* | *SE* | Minimum | Maximum |
| --- | --- | --- | --- | --- | --- |
|  | Foil Items (*N* = 15) | | | | |
| Homogeneous | 1.82 | 0.42 | 0.12 | 1.38 | 2.92 |
| Heterogeneous | 1.48 | 0.35 | 0.10 | 1.12 | 2.32 |
| Difference | 0.34 | 0.14 | 0.04 | 0.13 | 0.60 |
|  | Critical Specific Name Items (*N* = 15) | | | | |
| Homogeneous | 2.13 | 0.43 | 0.12 | 1.65 | 3.22 |
| Heterogeneous | 1.72 | 0.35 | 0.10 | 1.37 | 2.67 |
| Difference | 0.42 | 0.46 | 0.13 | -0.52 | 1.13 |
|  | Noncritical Specific Name Items (*N* = 45) | | | | |
| Homogeneous | 2.41 | 0.41 | 0.12 | 1.97 | 3.41 |
| Heterogeneous | 1.59 | 0.31 | 0.09 | 1.27 | 2.42 |
| Difference | 0.82 | 0.35 | 0.10 | 0.31 | 1.44 |
|  | Foil (*N* = 15) and Critical Specific Name Items (*N* = 15) | | | | |
| Homogeneous | 1.98 | 0.35 | 0.10 | 1.61 | 2.77 |
| Heterogeneous | 1.60 | 0.34 | 0.10 | 1.24 | 2.49 |
| Difference | 0.38 | 0.23 | 0.07 | 0.04 | 0.78 |

*Note.* Values on 5-point Likert scale. 1 = visually hardly similar; 5 = visually very similar.

**RESULTS FROM GLMM ANALYSES ON NONTRANSFORMED NAMING LATENCIES AND ERRORS (COMPLEMENTING THE ANALYSES REPORTED IN THE ARTICLE)**

Table S8a

*Model Implied Mean Naming Latencies (in ms) by Taxonomic Level, Context, and Cycle from Experiment 2. The Standard Error of the Mean is Given in Parentheses.*

| Context | Cycle | | | | | |  | Overall |
| --- | --- | --- | --- | --- | --- | --- | --- | --- |
|  | C_1_ | C_2_ | C_3_ | C_4_ | C_5_ | C_6_ |  | C_1_ – C_6_ |
| Specific Name | | | | | | | | |
| Homogeneous | 711  (13) | 652  (13) | 642  (11) | 654  (21) | 652  (12) | 653  (15) |  | 662  (5) |
| Heterogeneous | 735  (14) | 617  (14) | 602  (10) | 601  (20) | 604  (12) | 595  (12) |  | 626  (5) |
| Difference | -25  (10) | 35  (8) | 40  (8) | 52  (12) | 48  (8) | 58  (9) |  | 36  (3) |
| 95% CI | [-45; -5] | [18; 51] | [25; 55] | [28; 76] | [33; 63] | [40; 77] |  | [30; 41] |
| Foil | | | | | | | | |
| Homogeneous | 634  (14) | 588  (14) | 581  (10) | 590  (22) | 585  (12) | 589  (14) |  | 593  (5) |
| Heterogeneous | 634  (14) | 580  (15) | 573  (11) | 580  (21) | 572  (12) | 570  (14) |  | 585  (6) |
| Difference | 1  (12) | 8  (9) | 8  (6) | 10  (11) | 13  (7) | 19  (10) |  | 8  (3) |
| 95% CI | [-23; 24] | [-10; 25] | [-4; 21] | [-12; 31] | [0; 26] | [0; 38] |  | [3; 13] |

*Note.* CI = confidence interval.

Table S8b

*Model Implied Errors (in Log Odds) by Taxonomic Level, Context, and Cycle from Experiment 2. The Standard Error of the Mean is Given in Parentheses.*

| Context | Cycle | | | | | |  | Overall |
| --- | --- | --- | --- | --- | --- | --- | --- | --- |
|  | C_1_ | C_2_ | C_3_ | C_4_ | C_5_ | C_6_ |  | C_1_ – C_6_ |
| Specific Name | | | | | | | | |
| Homogeneous | -5.01  (0.62) | -5.53  (0.83) | -6.54  (1.51) | -4.82  (0.72) | -5.80  (1.11) | -5.55  (0.88) |  | -5.04  (0.40) |
| Heterogeneous | -5.07  (0.62) | -5.88  (0.87) | -8.78  (1.89) | -7.19  (1.56) | -7.96  (1.51) | -7.14  (1.12) |  | -5.99  (0.46) |
| Difference | 0.06  (0.50) | 0.35  (0.60) | 2.24  (1.10) | 2.37  (1.52) | 2.17  (1.08) | 1.59  (0.80) |  | 0.94  (0.31) |
| 95% CI | [-0.92; 1.04] | [-0.82; 1.52] | [0.09; 4.39] | [-0.61; 5.34] | [0.06; 4.27] | [0.02; 3.16] |  | [0.34; 1.54] |
| Foil | | | | | | | | |
| Homogeneous | -6.24  (0.83) | -10.34  (2.31) | -10.26  (2.60) | -8.02  (2.23) | -8.27  (2.49) | -6.85  (1.54) |  | -7.82  (0.73) |
| Heterogeneous | -5.93  (0.74) | -10.00  (2.27) | -10.74  (2.67) | -8.46  (2.69) | -7.71  (2.43) | -7.09  (1.57) |  | -7.67  (0.72) |
| Difference | -0.31  (0.74) | -0.34  (0.83) | 0.48  (1.00) | 0.44  (1.44) | -0.56  (0.76) | 0.24  (0.69) |  | -0.15  (0.33) |
| 95% CI | [-1.76; 1.15] | [-1.96; 1.28] | [-1.47; 2.44] | [-2.39; 3.26] | [-2.06; 0.94] | [-1.12; 1.60] |  | [-0.79; 0.49] |

*Note.* CI = confidence interval.

Table S9a

*Fixed Effects Estimates Based on GLMM Analyses for Naming Latencies (Nontransformed, Assuming a Gamma Distribution) and for Errors (Assuming a Binomial Distribution) across all Cycles from Experiment 2.*

|  | Naming Latencies | | | | | Errors | | | |
| --- | --- | --- | --- | --- | --- | --- | --- | --- | --- |
| Effect | Estimate | *SE* | *z* | *p* | Estimate | | *SE* | *z* | *p* |
| Intercept | 616 | 5 | 135.41 | < .001 | -6.63 | | 0.43 | -15.24 | < .001 |
| Homogeneous – Heterogeneous (C) | 22 | 2 | 9.21 | < .001 | 0.40 | | 0.22 | 1.77 | .076 |
| Foil – Specific Name  (L) | -54 | 3 | -15.71 | < .001 | -2.23 | | 0.75 | -2.96 | .003 |
| C:L | -28 | 3 | -9.18 | < .001 | -1.09 | | 0.45 | -2.44 | .015 |

*Note*. Formula for the analysis of naming latencies: RT ~ C + L + C:L + (C + L + C:L | participant) + (C || item). Formula for the analysis of errors: ER ~ C + L + C:L + (L | participant) + (C || item). C = semantic context; L = taxonomic level; RT = naming latency; ER = error.

Table S9b

*Fixed Effects Estimates Based on GLMM Analyses for Naming Latencies (Nontransformed, Assuming a Gamma Distribution) and for Errors (Assuming a Binomial Distribution) for Cycle 1 from Experiment 2.*

|  | Naming Latencies | | | | | Errors | | | |
| --- | --- | --- | --- | --- | --- | --- | --- | --- | --- |
| Effect | Estimate | *SE* | *z* | *p* | Estimate | | *SE* | *z* | *p* |
| Intercept | 679 | 12 | 58.22 | < .001 | -5.56 | | 0.55 | -10.04 | < .001 |
| Homogeneous – Heterogeneous (C) | -12 | 9 | -1.33 | .183 | -0.12 | | 0.48 | -0.26 | .798 |
| Foil – Specific Name  (L) | -89 | 9 | -9.59 | < .001 | -1.04 | | 0.62 | -1.69 | .091 |
| C:L | 26 | 13 | 1.99 | .046 | -0.37 | | 0.83 | -0.44 | .657 |

*Note*. Formula for the analysis of naming latencies: RT ~ C + L + C:L + (C + L + C:L | participant) + (C | item). Formula for the analysis of errors: ER ~ C + L + C:L + (C + L || participant) + (C || item). C = semantic context; L = taxonomic level; RT = naming latency; ER = error.

Table S9c

*Fixed Effects Estimates Based on GLMM Analyses for Naming Latencies (Nontransformed, Assuming a Gamma Distribution) and for Errors (Assuming a Binomial Distribution) for Cycle 2 from Experiment 2.*

|  | Naming Latencies | | | | | Errors | | | |
| --- | --- | --- | --- | --- | --- | --- | --- | --- | --- |
| Effect | Estimate | *SE* | *z* | *p* | Estimate | | *SE* | *z* | *p* |
| Intercept | 609 | 12 | 52.84 | < .001 | -7.94 | | 1.22 | -6.48 | < .001 |
| Homogeneous – Heterogeneous (C) | 21 | 6 | 3.28 | .001 | 0.01 | | 0.51 | 0.01 | .992 |
| Foil – Specific Name  (L) | -50 | 13 | -3.76 | < .001 | -4.47 | | 2.32 | -1.93 | .054 |
| C:L | -27 | 11 | -2.36 | .018 | -0.69 | | 1.02 | -0.67 | .500 |

*Note*. Formula for the analysis of naming latencies: RT ~ C + L + C:L + (C + L + C:L || participant) + (C | item). Formula for the analysis of errors: ER ~ C + L + C:L + (L | participant) + (1 | item). C = semantic context; L = taxonomic level; RT = naming latency; ER = error.

Table S9d

*Fixed Effects Estimates Based on GLMM Analyses for Naming Latencies (Nontransformed, Assuming a Gamma Distribution) and for Errors (Assuming a Binomial Distribution) for Cycle 3 from Experiment 2.*

|  | Naming Latencies | | | | | Errors | | | |
| --- | --- | --- | --- | --- | --- | --- | --- | --- | --- |
| Effect | Estimate | *SE* | *z* | *p* | Estimate | | *SE* | *z* | *p* |
| Intercept | 600 | 9 | 67.57 | < .001 | -9.08 | | 1.65 | -5.50 | < .001 |
| Homogeneous – Heterogeneous (C) | 24 | 4 | 5.39 | < .001 | 1.36 | | 0.74 | 1.84 | .066 |
| Foil – Specific Name  (L) | -45 | 10 | -4.72 | < .001 | -2.85 | | 2.78 | -1.02 | .306 |
| C:L | -31 | 11 | -2.93 | .003 | -1.76 | | 1.48 | -1.19 | .235 |

*Note*. Formula for the analysis of naming latencies: RT ~ C + L + C:L + (C + L + C:L | participant) + (C | item). Formula for the analysis of errors: ER ~ C + L + C:L + (L | participant) + (1 | item). C = semantic context; L = taxonomic level; RT = naming latency; ER = error.

Table S9e

*Fixed Effects Estimates Based on GLMM Analyses for Naming Latencies (Nontransformed, Assuming a Gamma Distribution) and for Errors (Assuming a Binomial Distribution) for Cycle 4 from Experiment 2.*

|  | Naming Latencies | | | | | Errors | | | |
| --- | --- | --- | --- | --- | --- | --- | --- | --- | --- |
| Effect | Estimate | *SE* | *z* | *p* | Estimate | | *SE* | *z* | *p* |
| Intercept | 606 | 20 | 30.93 | < .001 | -7.12 | | 1.41 | -5.04 | < .001 |
| Homogeneous – Heterogeneous (C) | 31 | 9 | 3.64 | < .001 | 1.40 | | 1.33 | 1.06 | .291 |
| Foil – Specific Name  (L) | -43 | 11 | -3.85 | < .001 | -2.23 | | 2.24 | -1.00 | .318 |
| C:L | -43 | 16 | -2.70 | .007 | -1.93 | | 1.31 | -1.47 | .141 |

*Note*. Formula for the analysis of naming latencies: RT ~ C + L + C:L + (C + L + C:L || participant) + (C | item). Formula for the analysis of errors: ER ~ C + L + C:L + (L | participant) + (C | item). C = semantic context; L = taxonomic level; RT = naming latency; ER = error.

Table S9f

*Fixed Effects Estimates Based on GLMM Analyses for Naming Latencies (Nontransformed, Assuming a Gamma Distribution) and for Errors (Assuming a Binomial Distribution) for Cycle 5 from Experiment 2.*

|  | Naming Latencies | | | | | Errors | | | |
| --- | --- | --- | --- | --- | --- | --- | --- | --- | --- |
| Effect | Estimate | *SE* | *z* | *p* | Estimate | | *SE* | *z* | *p* |
| Intercept | 603 | 11 | 56.91 | < .001 | -7.44 | | 1.45 | -5.11 | < .001 |
| Homogeneous – Heterogeneous (C) | 30 | 6 | 5.09 | < .001 | 0.80 | | 0.66 | 1.22 | .223 |
| Foil – Specific Name  (L) | -50 | 8 | -6.08 | < .001 | -1.11 | | 2.51 | -0.44 | .657 |
| C:L | -35 | 8 | -4.58 | < .001 | -2.72 | | 1.32 | -2.07 | .039 |

*Note*. Formula for the analysis of naming latencies: RT ~ C + L + C:L + (C + L + C:L | participant) + (C || item). Formula for the analysis of errors: ER ~ C + L + C:L + (L | participant) + (1 | item). C = semantic context; L = taxonomic level; RT = naming latency; ER = error.

Table S9g

*Fixed Effects Estimates Based on GLMM Analyses for Naming Latencies (Nontransformed, Assuming a Gamma Distribution) and for Errors (Assuming a Binomial Distribution) for Cycle 6 from Experiment 2.*

|  | Naming Latencies | | | | | Errors | | | |
| --- | --- | --- | --- | --- | --- | --- | --- | --- | --- |
| Effect | Estimate | *SE* | *z* | *p* | Estimate | | *SE* | *z* | *p* |
| Intercept | 602 | 12 | 48.91 | < .001 | -6.66 | | 0.95 | -7.02 | < .001 |
| Homogeneous – Heterogeneous (C) | 39 | 7 | 5.22 | < .001 | 0.92 | | 0.53 | 1.73 | .084 |
| Foil – Specific Name  (L) | -44 | 8 | -5.21 | < .001 | -0.63 | | 1.64 | -0.38 | .702 |
| C:L | -39 | 12 | -3.36 | .001 | -1.35 | | 1.06 | -1.28 | .202 |

*Note*. Formula for the analysis of naming latencies: RT ~ C + L + C:L + (C + L + C:L | participant) + (C | item). Formula for the analysis of errors: ER ~ C + L + C:L + (L | participant) + (1 | item). C = semantic context; L = taxonomic level; RT = naming latency; ER = error.

**RESULTS FROM LMM ANALYSES ON INVERSE NAMING LATENCIES (ADDITIONAL ANALYSES)**

Table S10a

*Fixed Effects Estimates Based on LMM Analyses for Inverse Naming Latencies (- 1000/RT, Assuming a Normal Distribution) across all Cycles from Experiment 2.*

| Effect | Estimate | *SE* | *df* | *t* | *p* |
| --- | --- | --- | --- | --- | --- |
| Intercept | -1.72 | 0.03 | 62.01 | -66.64 | < .001 |
| Homogeneous – Heterogeneous (C) | 0.06 | 0.01 | 39.04 | 7.42 | < .001 |
| Foil – Specific Name  (L) | -0.14 | 0.03 | 34.41 | -4.86 | < .001 |
| C:L | -0.06 | 0.01 | 25.59 | -4.50 | < .001 |

*Note*. Formula: RT ~ C + L + C:L + (C + L + C:L || participant) + (C | item). C = semantic context; L = taxonomic level; RT = inverse naming latency.

Table S10b

*Fixed Effects Estimates Based on LMM Analyses for Inverse Naming Latencies (- 1000/RT, Assuming a Normal Distribution) for Cycle 1 from Experiment 2.*

| Effect | Estimate | *SE* | *df* | *t* | *p* |
| --- | --- | --- | --- | --- | --- |
| Intercept | -1.58 | 0.03 | 61.77 | -62.85 | < .001 |
| Homogeneous – Heterogeneous (C) | -0.02 | 0.02 | 46.60 | -1.09 | .283 |
| Foil – Specific Name  (L) | -0.20 | 0.04 | 35.23 | -5.25 | < .001 |
| C:L | 0.03 | 0.05 | 46.56 | 0.69 | .491 |

*Note*. Formula: RT ~ C + L + C:L + (C + L + C:L | participant) + (C | item). C = semantic context; L = taxonomic level; RT = inverse naming latency.

Table S10c

*Fixed Effects Estimates Based on LMM Analyses for Inverse Naming Latencies (- 1000/RT, Assuming a Normal Distribution) for Cycle 2 from Experiment 2.*

| Effect | Estimate | *SE* | *df* | *t* | *p* |
| --- | --- | --- | --- | --- | --- |
| Intercept | -1.72 | 0.02 | 59.51 | -71.46 | < .001 |
| Homogeneous – Heterogeneous (C) | 0.05 | 0.01 | 29.83 | 5.30 | < .001 |
| Foil – Specific Name  (L) | -0.13 | 0.03 | 33.21 | -4.57 | < .001 |
| C:L | -0.06 | 0.02 | 29.83 | -2.74 | .010 |

*Note*. Formula: RT ~ C + L + C:L + (L | participant) + (C | item). C = semantic context; L = taxonomic level; RT = inverse naming latency.

Table S10d

*Fixed Effects Estimates Based on LMM Analyses for Inverse Naming Latencies (- 1000/RT, Assuming a Normal Distribution) for Cycle 3 from Experiment 2.*

| Effect | Estimate | *SE* | *df* | *t* | *p* |
| --- | --- | --- | --- | --- | --- |
| Intercept | -1.75 | 0.03 | 59.59 | -68.04 | < .001 |
| Homogeneous – Heterogeneous (C) | 0.06 | 0.01 | 2,229.80 | 6.18 | < .001 |
| Foil – Specific Name  (L) | -0.12 | 0.03 | 29.81 | -4.33 | < .001 |
| C:L | -0.06 | 0.02 | 2,229.78 | -2.94 | .003 |

*Note*. Formula: RT ~ C + L + C:L + (L || participant) + (1 | item). C = semantic context; L = taxonomic level; RT = inverse naming latency.

Table S10e

*Fixed Effects Estimates Based on LMM Analyses for Inverse Naming Latencies (- 1000/RT, Assuming a Normal Distribution) for Cycle 4 from Experiment 2.*

| Effect | Estimate | *SE* | *df* | *t* | *p* |
| --- | --- | --- | --- | --- | --- |
| Intercept | -1.74 | 0.03 | 52.49 | -63.78 | < .001 |
| Homogeneous – Heterogeneous (C) | 0.07 | 0.01 | 21.55 | 5.96 | < .001 |
| Foil – Specific Name  (L) | -0.12 | 0.02 | 29.36 | -4.78 | < .001 |
| C:L | -0.09 | 0.02 | 30.39 | -3.62 | .001 |

*Note*. Formula: RT ~ C + L + C:L + (C || participant) + (C | item). C = semantic context; L = taxonomic level; RT = inverse naming latency.

Table S10f

*Fixed Effects Estimates Based on LMM Analyses for Inverse Naming Latencies (- 1000/RT, Assuming a Normal Distribution) for Cycle 5 from Experiment 2.*

| Effect | Estimate | *SE* | *df* | *t* | *p* |
| --- | --- | --- | --- | --- | --- |
| Intercept | -1.76 | 0.03 | 57.20 | -61.47 | < .001 |
| Homogeneous – Heterogeneous (C) | 0.08 | 0.01 | 39.64 | 6.66 | < .001 |
| Foil – Specific Name  (L) | -0.13 | 0.03 | 29.38 | -4.46 | < .001 |
| C:L | -0.08 | 0.02 | 2,230.66 | -4.04 | < .001 |

*Note*. Formula: RT ~ C + L + C:L + (C | participant) + (1 | item). C = semantic context; L = taxonomic level; RT = inverse naming latency.

Table S10g

*Fixed Effects Estimates Based on LMM Analyses for Inverse Naming Latencies (- 1000/RT, Assuming a Normal Distribution) for Cycle 6 from Experiment 2.*

| Effect | Estimate | *SE* | *df* | *t* | *p* |
| --- | --- | --- | --- | --- | --- |
| Intercept | -1.76 | 0.03 | 53.80 | -62.76 | < .001 |
| Homogeneous – Heterogeneous (C) | 0.10 | 0.01 | 29.89 | 6.57 | < .001 |
| Foil – Specific Name  (L) | -0.11 | 0.03 | 30.09 | -4.32 | < .001 |
| C:L | -0.08 | 0.02 | 29.97 | -3.76 | .001 |

*Note*. Formula: RT ~ C + L + C:L + (C + L || participant) + (C | item). C = semantic context; L = taxonomic level; RT = inverse naming latency.

**RESULTS FROM ANOVAS ON NAMING LATENCIES AND ERROR PROPORTIONS (ADDITIONAL ANALYSES)**

Table S11a

*Mean Naming Latencies (in ms) by Taxonomic Level, Context, and Cycle from Experiment 2. The Standard Error of the Mean is Given in Parentheses.*

| Context | Cycle | | | | | |  | Overall |
| --- | --- | --- | --- | --- | --- | --- | --- | --- |
|  | C_1_ | C_2_ | C_3_ | C_4_ | C_5_ | C_6_ |  | C_1_ – C_6_ |
| Specific Name | | | | | | | | |
| Homogeneous | 694  (8) | 644  (10) | 633  (9) | 642  (11) | 638  (12) | 638  (12) |  | 648  (9) |
| Heterogeneous | 717  (11) | 608  (8) | 594  (7) | 589  (10) | 590  (12) | 581  (10) |  | 613  (9) |
| Difference | -23  (9) | 36  (7) | 39  (6) | 53  (7) | 48  (7) | 57  (8) |  | 35  (3) |
| 95% CI | [-41; -5] | [22; 50] | [27; 52] | [39; 66] | [34; 62] | [42; 72] |  | [29; 41] |
| Foil | | | | | | | | |
| Homogeneous | 614  (13) | 578  (8) | 572  (8) | 576  (9) | 572  (11) | 577  (9) |  | 581  (8) |
| Heterogeneous | 614  (11) | 571  (9) | 563  (10) | 569  (13) | 559  (10) | 558  (10) |  | 572  (10) |
| Difference | 0  (15) | 7  (5) | 9  (5) | 7  (7) | 13  (6) | 19  (6) |  | 9  (3) |
| 95% CI | [-30; 30] | [-3; 18] | [-1; 18] | [-6; 20] | [2; 24] | [7; 31] |  | [3; 16] |

*Note.* CI = confidence interval. Naming latencies are based on aggregated participant data.

Table S11b

*Mean Error proportions (in %) by Taxonomic Level, Context, and Cycle from Experiment 2. The Standard Error of the Mean is Given in Parentheses.*

| Context | Cycle | | | | | |  | Overall |
| --- | --- | --- | --- | --- | --- | --- | --- | --- |
|  | C_1_ | C_2_ | C_3_ | C_4_ | C_5_ | C_6_ |  | C_1_ – C_6_ |
| Specific Name | | | | | | | | |
| Homogeneous | 2.17  (0.60) | 1.17  (0.47) | 1.33  (0.49) | 2.00  (0.59) | 1.33  (0.49) | 1.50  (0.56) |  | 1.58  (0.34) |
| Heterogeneous | 2.00  (0.64) | 0.83  (0.35) | 0.17  (0.17) | 0.50  (0.28) | 0.17  (0.17) | 0.33  (0.23) |  | 0.67  (0.19) |
| Difference | 0.17  (0.91) | 0.33  (0.53) | 1.17  (0.41) | 1.50  (0.56) | 1.17  (0.47) | 1.17  (0.53) |  | 0.92  (0.28) |
| 95% CI | [-1.67; 2.00] | [-0.74; 1.41] | [0.35; 1.99] | [0.37; 2.63] | [0.21; 2.12] | [0.10; 2.23] |  | [0.35; 1.49] |
| Foil | | | | | | | | |
| Homogeneous | 0.67  (0.40) | 0.50  (0.28) | 0.50  (0.28) | 0.50  (0.28) | 0.50  (0.28) | 0.83  (0.35) |  | 0.58  (0.27) |
| Heterogeneous | 0.83  (0.35) | 0.67  (0.32) | 0.33  (0.23) | 0.67  (0.32) | 0.83  (0.35) | 0.67  (0.32) |  | 0.67  (0.27) |
| Difference | -0.17  (0.29) | -0.17  (0.17) | 0.17  (0.17) | -0.17  (0.29) | -0.33  (0.23) | 0.17  (0.29) |  | -0.08  (0.09) |
| 95% CI | [-0.76; 0.42] | [-0.50; 0.17] | [-0.17; 0.50] | [-0.76; 0.42] | [-0.80; 0.14] | [-0.42; 0.76] |  | [-0.27; 0.10] |

*Note.* CI = confidence interval. Error proportions are based on aggregated participant data.

Table S12

*Analyses of Variance for Naming Latencies and Error Proportions including factors Context, Cycle, and Taxonomic Level from Experiment 2.*

| Participant Analysis | | | | | Item Analysis | | | | |
| --- | --- | --- | --- | --- | --- | --- | --- | --- | --- |
| Effect | df | *F* | *p* | η_G_^2^ | Effect | df | *F* | *p* | η_G_^2^ |
| Naming Latencies | | | | | | | | | |
| C | 1, 39 | 85.08 | < .001 | .030 | C | 1, 28 | 58.99 | < .001 | .093 |
| Cy | 2.49, 97.24 | 92.18 | < .001 | .138 | Cy | 2.15, 60.29 | 88.10 | < .001 | .346 |
| C:Cy | 2.53, 98.59 | 11.44 | < .001 | .015 | C:Cy | 3.02, 84.59 | 12.41 | < .001 | .045 |
| L | 1, 39 | 232.86 | < .001 | .154 | L | 1, 28 | 24.08 | < .001 | .368 |
| C:L | 1, 39 | 39.62 | < .001 | .010 | C:L | 1, 28 | 19.06 | < .001 | .032 |
| Cy:L | 3.48, 135.56 | 20.72 | < .001 | .018 | Cy:L | 2.15, 60.29 | 9.89 | < .001 | .056 |
| C:Cy:L | 2.76, 107.60 | 4.90 | .004 | .008 | C:Cy:L | 3.02, 84.59 | 6.41 | .001 | .024 |
| Error Proportions | | | | | | | | | |
| C | 1, 39 | 7.60 | .009 | .007 | C | 1, 28 | 7.32 | .011 | .015 |
| Cy | 3.35, 130.53 | 3.46 | .015 | .012 | Cy | 2.67, 74.78 | 2.86 | .048 | .024 |
| C:Cy | 2.89, 112.58 | 0.86 | .462 | .003 | C:Cy | 3.15, 88.07 | 0.85 | .475 | .007 |
| L | 1, 39 | 4.03 | .052 | .010 | L | 1, 28 | 1.37 | .252 | .022 |
| C:L | 1, 39 | 11.97 | .001 | .010 | C:L | 1, 28 | 10.54 | .003 | .022 |
| Cy:L | 3.78, 147.59 | 2.26 | .069 | .007 | Cy:L | 2.67, 74.78 | 1.79 | .163 | .015 |
| C:Cy:L | 3.01, 117.42 | 0.84 | .473 | .002 | C:Cy:L | 3.15, 88.07 | 0.62 | .608 | .005 |

*Note.* Corrected degrees of freedom (df) and *p*-values are reported (based on Greenhouse-Geisser correction), if the sphericity assumption is violated (Mauchly's test: *p* < .05).C = semantic context; Cy = cycle; L = taxonomic level.

**RESULTS FROM CROSSEXPERIMENTAL (G)LMM ANALYSES ON NONTRANSFORMED AND INVERSE NAMING LATENCIES (ADDITIONAL ANALYSES)**

Table S13a

*Fixed Effects Estimates Based on (G)LMM Analyses for Nontransformed Naming Latencies (Assuming a Gamma Distribution) and for Inverse Naming Latencies (Assuming a Normal Distribution) across all Cycles from Experiments 1 and 2.*

|  | Nontransformed  Naming Latencies | | | | Inverse  Naming Latencies | | | | |
| --- | --- | --- | --- | --- | --- | --- | --- | --- | --- |
|  | Estimate | *SE* | *z* | *p* | Estimate | *SE* | *df* | *t* | *p* |
| Intercept | 620 | 1 | 426.28 | < .001 | -1.71 | 0.02 | 113.48 | -88.11 | < .001 |
| Homogeneous – Heterogeneous (C) | 28 | 2 | 16.35 | < .001 | 0.07 | 0.01 | 78.79 | 10.56 | < .001 |
| Alternative Name / Foil –  Specific Name  (L) | -47 | 2 | -28.56 | < .001 | -0.12 | 0.02 | 62.83 | -6.65 | < .001 |
| Experiment 1 –  Experiment 2 (E) | 20 | 2 | 12.76 | < .001 | 0.01 | 0.04 | 99.89 | 0.32 | .747 |
| C:L | -14 | 3 | -4.91 | < .001 | -0.03 | 0.01 | 56.36 | -2.46 | .017 |
| C:E | 10 | 3 | 3.67 | < .001 | 0.02 | 0.01 | 100.03 | 1.99 | .049 |
| L:E | 4 | 2 | 1.90 | .057 | 0.02 | 0.03 | 86.29 | 0.90 | .372 |
| C:L:E | 24 | 2 | 11.62 | < .001 | 0.05 | 0.02 | 77.62 | 2.57 | .012 |

*Note*. Formula for the analysis of nontransformed naming latencies: RT ~ C + L + E + C:L + C:E + L:E + C:L:E + (C + L + C:L | participant) + (C | item). Formula for the analysis of inverse naming latencies: RT ~ C + L + E + C:L + C:E + L:E + C:L:E + (C + L + C:L || participant) + (C | item). C = semantic context; L = taxonomic level; E = experiment; RT = (nontransformed/inverse) naming latency.

Table S13b

*Fixed Effects Estimates Based on (G)LMM Analyses for Nontransformed Naming Latencies (Assuming a Gamma Distribution) and for Inverse Naming Latencies (Assuming a Normal Distribution) for Specific Name Responses across all Cycles from Experiments 1 and 2.*

|  | Nontransformed  Naming Latencies | | | | Inverse  Naming Latencies | | | | |
| --- | --- | --- | --- | --- | --- | --- | --- | --- | --- |
|  | Estimate | *SE* | *z* | *p* | Estimate | *SE* | *df* | *t* | *p* |
| Intercept | 644 | 3 | 226.13 | < .001 | -1.65 | 0.02 | 92.30 | -76.88 | < .001 |
| Homogeneous – Heterogeneous (C) | 34 | 2 | 16.77 | < .001 | 0.08 | 0.01 | 32.97 | 8.53 | < .001 |
| Experiment 1 – Experiment 2  (E) | 20 | 3 | 7.63 | < .001 | < 0.01 | 0.03 | 89.88 | -0.01 | .994 |
| C:E | -3 | 2 | -1.19 | .236 | < 0.01 | 0.02 | 98.30 | -0.18 | .857 |

*Note*. Formula: RT ~ C + E + C:E + (C | participant) + (C | item). C = semantic context; E = experiment; RT = (nontransformed/inverse) naming latency.

Table S13c

*Fixed Effects Estimates Based on (G)LMM Analyses for Nontransformed Naming Latencies (Assuming a Gamma Distribution) and for Inverse Naming Latencies (Assuming a Normal Distribution) for Alternative Name / Foil Responses across all Cycles from Experiments 1 and 2.*

|  | Nontransformed  Naming Latencies | | | | Inverse  Naming Latencies | | | | |
| --- | --- | --- | --- | --- | --- | --- | --- | --- | --- |
|  | Estimate | *SE* | *z* | *p* | Estimate | *SE* | *df* | *t* | *p* |
| Intercept | 597 | 3 | 212.19 | < .001 | -1.77 | 0.02 | 105.62 | -81.88 | < .001 |
| Homogeneous – Heterogeneous (C) | 21 | 2 | 9.24 | < .001 | 0.05 | 0.01 | 43.33 | 7.18 | < .001 |
| Experiment 1 – Experiment 2  (E) | 16 | 2 | 7.30 | < .001 | 0.02 | 0.04 | 105.62 | 0.57 | .571 |
| C:E | 22 | 3 | 6.27 | < .001 | 0.05 | 0.02 | 43.33 | 3.35 | .002 |

*Note*. Formula: RT ~ C + E + C:E + (C | participant) + (C | item). C = semantic context; E = experiment; RT = (nontransformed/inverse) naming latency.

**SUPPLEMENTARY MATERIAL 2**

Appendix A1

*List of the picture names used, subset 1. English translations are given in brackets. Critical specific name items are marked by asterisks.*

| Set | Het 1 (mixed) | Het 2 (mixed) | Het 3 (mixed) | Het 4 (mixed) | Het 5 (mixed) |
| --- | --- | --- | --- | --- | --- |
| Hom 1 (fishes) | FISCH^1^ [fish] | Hai [shark] | *Aal [eel] | **Koi [koi] | Rochen [ray] |
| Hom 2 (cars) | Jeep [Jeep] | AUTO^2^ [car] | **Käfer [Beetle] | *Smart [Smart] | Porsche [Porsche] |
| Hom 3 (flowers) | Nelke [carnation] | Orchidee [orchid] | BLUME^3^ [flower] | Rose [rose] | *^/^**Tulpe [tulip] |
| Hom 4 (puppets) | *Barbie [Barbie] | **Marionette [marionette] | Matroschka [matryoshka] | PUPPE^4^ [puppet] | Kasper [punch] |
| Hom 5 (shoes) | **Sandale [sandal] | *Flipflop [flip-flop] | Pumps [pumps] | Ballerina [ballerina shoe] | SCHUH^5^ [shoe] |

*Note*. Capital letters indicate alternative name items; Hom = homogeneous; Het = heterogeneous; * = critical specific name item in Experiment 1; ** = critical specific name item in Experiment 2. Foil replacements for alternative name items in Experiment 2: ^1^ Spachtel [scraper], ^2^ Topf [pot], ^3^ Ball [ball], ^4^ Hydrant [fireplug], ^5^ Messer [knife].

Appendix A2

*List of the picture names used, subset 2. English translations are given in brackets. Critical specific name items are marked by asterisks.*

| Set | Het 6 (mixed) | Het 7 (mixed) | Het 8 (mixed) | Het 9 (mixed) | Het 10 (mixed) |
| --- | --- | --- | --- | --- | --- |
| Hom 6 (dogs) | HUND^1^ [dog] | *Mops [pug] | Dackel [dachshund] | **Pudel [poodle] | Collie [collie] |
| Hom 7 (cheese) | Parmesan [parmesan] | KÄSE^2^ [cheese] | Feta [feta] | Brie [brie] | *^/^**Mozzarella [mozzarella] |
| Hom 8 (trousers) | **Jeans [jeans] | Baggy [baggy pants] | HOSE^3^ [trousers] | *Leggings [leggings] | Shorts [shorts] |
| Hom 9 (pens) | *Kuli [ballpoint pen] | **Edding [permanent marker] | Marker [marker] | STIFT^4^ [pen] | Füller [fountain pen] |
| Hom 10 (trees) | Eiche [oak] | Weide [willow] | *^/^**Palme [palm tree] | Tanne [fir] | BAUM^5^ [tree] |

*Note*. Capital letters indicate alternative name items; Hom = homogeneous; Het = heterogeneous; * = critical specific name item in Experiment 1; ** = critical specific name item in Experiment 2.. Foil replacements for alternative name items in Experiment 2: ^1^ Stuhl [chair], ^2^ Iglu [igloo], ^3^ Zirkel [compass], ^4^ Zigarette [cigarette], ^5^ Lampe [lamp].

Appendix A3

*List of the picture names used, subset 3. English translations are given in brackets. Critical specific name items are marked by asterisks.*

| Set | Het 11 (mixed) | Het 12 (mixed) | Het 13 (mixed) | Het 14 (mixed) | Het 15 (mixed) |
| --- | --- | --- | --- | --- | --- |
| Hom 11 (birds) | VOGEL^1^ [bird] | Adler [eagle] | Ente [duck] | **Storch [stork] | *Papagei [parrot] |
| Hom 12 (breads) | Sandwich [sandwich] | BROT^2^ [bread] | *Toast [toast] | Pumpernickel [pumpernickel] | **Ciabatta [ciabatta] |
| Hom 13 (athletes) | **Boxer [boxer] | Fußballer [footballer] | SPORTLER^3^ [athlete] | *Golfer [golfer] | Turner [gymnast] |
| Hom 14 (cards) | *Ass [ace] | **Joker [joker] | Bube [knave] | KARTE^4^ [card] | Dame [queen] |
| Hom 15 (books) | Koran [Koran] | *Duden [dictionary] | **Atlas [atlas] | Lexikon [lexicon] | BUCH^5^ [book] |

*Note*. Capital letters indicate alternative name items; Hom = homogeneous; Het = heterogeneous; * = critical specific name item in Experiment 1; ** = critical specific name item in Experiment 2. Foil replacements for alternative name items in Experiment 2: ^1^ Gießkanne [watering can], ^2^ Kissen [pillow], ^3^ Kreuz [cross], ^4^ Turm [tower], ^5^ Eimer [bucket].
